# Comprehensive Analysis of the Prognostic and Immunological Role of PAFAH1B in Pan-Cancer

**DOI:** 10.3389/fmolb.2021.799497

**Published:** 2022-02-03

**Authors:** Yixiao Yuan, Xiulin Jiang, Lin Tang, Juan Wang, Lincan Duan

**Affiliations:** ^1^ Department of Thoracic Surgery, The Third Affiliated Hospital of Kunming Medical University, Kunming, China; ^2^ Key Laboratory of Animal Models and Human Disease Mechanisms of Chinese Academy of Sciences and Yunnan Province, Kunming Institute of Zoology, Kunming, China

**Keywords:** PAFAH1B3, pan-cancer, immune cell infiltration, drug sensitivity, prognostic biomarker, multi-omics integrative analysis, cell proliferation, cell migration

## Abstract

Platelet-activating factor acetylhydrolase 1B3 (PAFAH1B3) plays a critical role in cancer initiation, metastasis, and progression; however, it remains unknown how PAFAH1B3 impacts cancer diagnosis and prognosis or regulates the immune response to different types of cancer. In this study, PAFAH1B3 was elevated in human pan-cancer, and this correlated with greater pathology and poor prognosis, in particular for non-small cell lung cancer (NSCLC) and liver hepatocellular carcinoma (LIHC). In addition, PAFAH1B3 expression was positively associated with tumor mutational burden (TMB), microsatellite instability (MSI), immune cell infiltration, immune-modulatory related gene expression, and diverse cancer drug sensitivity in human cancer. Increased PAFAH1B3 expression correlated with poor overall survival (OS), disease-specific survival (DSS), and progression-free interval (PFI) of NSCLC and LIHC, and has potential as an independent risk factor for overall survival (OS), disease-specific survival (DSS), and progression-free interval (PFI) during LIHC. Kyoto Encyclopedia of Genes and Genomes (KEGG) enrichment analysis confirmed that PAFAH1B3 is primarily involved in immune regulation. More importantly, results demonstrated that PAFAH1B3 was upregulated in liver cancer cells lines and that knockdown of this gene significantly inhibited cell proliferation, migration, and invasion in liver hepatocellular carcinoma (LIHC). In summary, this study elucidates the clinical significance and biological function of PAFAH1B3 during liver hepatocellular carcinoma (LIHC) and may serve as a potential biomarker for the diagnosis and prognosis of various cancer types.

## Introduction

Cancer affects millions of people each year and poses a substantial societal and economic burden worldwide. Despite available surgical and chemotherapeutic treatment modalities, cancer prognosis often remains poor. Cancer hallmark genes (e.g., BRCA1, CDK1, E2F1, and EGFR) are responsible for the most essential phenotypic characteristics of malignant transformation and progression (Nagy et al., 2021). Thus, there is an urgent need to identify specific molecular targets to improve cancer diagnosis and treatment.

Platelet-activating factor acetylhydrolase 1B3 (PAFAH1B3), one of the catalytic subunits of PAFAH, plays an important role in apoptosis (Monillas et al., 2015), cancer metastasis (Stafforini, 2015), and angiogenesis during cancer (Sun et al., 2015). PAFAH1B3 is involved in diverse cancer-related signaling pathways, including PAF and WNT, and facilitates cancer progression (Manya et al., 1999; Livnat et al., 2010). By modulating tumor-suppressing lipids, PAFAH1B3 promotes cancer cell aggressiveness (Kume and Shimizu, 1997). In addition, Michael et al. showed that PAFAH1B3 may be a potential target for tyrosine kinase inhibitors (TKIs) in breast cancer (BRCA) (Fiedler et al., 2018). PAFAH1B3 was found to play a crucial role in the brain development process. lissencephaly associated mutations will destroy the interaction between PAFAH1B3 and PAFAH1B2, leading to inhibitions in the neuronal migration (Xing et al., 2011). These studies indicate that PAFAH1B3 regulates diverse biological functions in cancer initiation, metastasis, and progression, and may be a promising prognostic and therapeutic biomarker for pan-cancer. However, the specific role of PAFAH1B3 in diagnosis, prognosis, and immune regulation in various types of cancer remains unexplored.

In this study, public databases were used for the first time to show that PAFAH1B3 is highly expressed in diverse cancer types. PAFAH1B3 expression correlates significantly with pathology and poor prognosis and is highly accurate at predicting cancer progression. In addition, PAFAH1B3 expression was positively associated with tumor mutational burden (TMB), microsatellite instability (MSI), immune cell infiltration, immune-modulatory related gene expression, and diverse cancer drug sensitivity in human cancer. Finally, high expression of PAFAH1B3 correlated with poor overall survival (OS), disease-specific survival (DSS), and progression-free interval (PFI) in non-small cell lung cancer (NSCLC) and liver hepatocellular carcinoma (LIHC), and has the potential as an independent risk factor for overall survival (OS), disease-specific survival (DSS), and progression-free interval (PFI) in liver hepatocellular carcinoma (LIHC). Kyoto Encyclopedia of Genes and Genomes (KEGG) enrichment analysis was used to confirm that PAFAH1B3 is primarily involved in immune regulation. More importantly, results demonstrated that PAFAH1B3 was up-regulated in liver hepatocellular carcinoma (LIHC) cells lines. Knockdown of PAFAH1B3 significantly inhibited cell proliferation, migration, and invasion in liver hepatocellular carcinoma (LIHC). In summary, PAFAH1B3 is a potential biomarker for diagnosis and prognosis in different cancer types and a promising molecular target for LIHC.

## Materials and Methods

### Analysis of the Expression of PAFAH1B3 in Pan-Cancer

We utilized TIMER (https://cistrome.shinyapps.io/timer/) (Li et al., 2017), TCGA (https://www.cancer.gov/about-nci/organization/ccg/research/structural-genomics/tcga), Genotype-Tissue Expression (GTEx) database, ualcan database (http://ualcan.path.uab.edu/) (Chandrashekar et al., 2017), and CCLE database (https://portals.broadinstitute.org/ccle/) (Ghandi et al., 2019) to examine the expression of PAFAH1B3 in pan-cancer tissue and cancer cells lines (ns, *p* ≥ 0.05; *, *p* < 0.05; **, *p* < 0.01; ***, *p* < 0.001).

### Analysis the Prognosis and Clinical Information of PAFAH1B3 in Pan-Cancer

We employed the GEPIA databases (http://gepia.cancer-pku.cn/) and prognoscan databases (http://dna00.bio.kyutech.ac.jp/PrognoScan/index.html) (Mizuno et al., 2009; Tang et al., 2017) to examine the OS and RFS of PAFAH1B3 in pan-cancer; additionally, the correlation between the pathology stage and PAFAH1B3 expression was analysis by GEPIA, the correlation between the tumor stage and PAFAH1B3 expression was analysis by GEPIA (ns, *p* ≥ 0.05; *, *p* < 0.05; **, *p* < 0.01; ***, *p* < 0.001).

### Analysis of the Gene Mutation of PAFAH1B3 in Pan-Cancer

The gene mutation information of PAFAH1B3 in pan-cancer was analyzed by Cerami et al. (2012).

### Analysis of the Function of PAFAH1B3 in Pan-Cancer

We utilized the cbioportal database (https://www.cbioportal.org/) to analyze the co-expression genes in pan-cancer. KEGG enrichment analyses was analysed by the cluster Profiler package and using ggplot2 package for visualization (Yu et al., 2012; Ito and Murphy, 2013).

### Analysis of the Immunological Functions of PAFAH1B3 in Pan-Cancer

We employed the TIMER (https://cistrome.shinyapps.io/timer/) and XCELL tools (https://xcell.ucsf.edu/) to analyze the immunological roles of PAFAH1B3 (Li et al., 2017; Aran et al., 2017), including the correlation between the diverse immune cells and immune regulator. The TISIDB (http://cis.hku.hk/TISIDB/) was utilized to analysis the expression of PAFAH1B3 in molecular subtypes and immune subtypes of diverse cancers (Ru et al., 2019). The TMB and MSI scores were obtained from TCGA. Correlation analysis between the PAFAH1B3 expression and TMB or MSI was performed using spearman’s methods (ns, *p* ≥ 0.05; *, *p* < 0.05; **, *p* < 0.01; ***, *p* < 0.001).

### Correlation Between PAFAH1B3 Expression and Cancer Drug Sensitivity

We utilized GDSC and CTRP databases to analyze the correlation between PAFAH1B3 expression and drug sensitivity (Basu et al., 2013; Yang et al., 2013).

### Cell Culture

The LO2 cell line was purchased from the cell bank of Kunming Institute of Zoology, and cultured in DMEM media (Lonza, CC-3170). Liver cancer cell lines, including HepG2, Hu7, and SMCC-7721, were purchased from Cobioer, China, with STR document, HepG2, Hu7, and SMCC-7721 cells were all cultured in DMEM medium (Corning) supplemented with 10% fetal bovine serum (FBS) and 1% penicillin/streptomycin. The siRNA for PAFAH1B3 were synthesized by RIBOBIO, and a scrambled siRNA was synthesized as a negative control. Transfection was performed using Lipofectamine 2000 (Invitrogen) according to the manufacturer’s instructions. Total RNA was collected 48 h after transfection.

### Quantitative Real-Time PCR

The qRT-PCR assay was performed as documented (Jiang et al., 2018). The primer sequences are as follows: PAFAH1B3-F: ACA​TCC​GGC​CCA​AGA​TTG​TG, PAFAH1B3-R: GGG​CTG​TCG​CTC​ATT​CAC​C, PAFAH1B1-F: TCT​TGG​TCA​GAA​ACG​AGA​CCC, PAFAH1B1-R: GTG​GTC​GAA​TGA​AAT​GTC​CTG​TA. PAFAH1B2-F: CAA​ACC​CAG​CAG​CTA​TTC​CG, PAFAH1B2-R: GAA​CAG​TAC​ATC​AGG​CTC​TTT​GT. BRCA1-F: GCT​CGT​GGA​AGA​TTT​CGG​TGT, BRCA1-R: TCA​TCA​ATC​ACG​GAC​GTA​TCA​TC. CDK1-F: ACC​GAA​GGG​AGA​ACG​ACG​AA, CDK1-R: GAA​CGC​TTT​GAA​CTT​CCC​GAT. E2F1-F: ACG​CTA​TGA​GAC​CTC​ACT​GAA, E2F1-R: TCC​TGG​GTC​AAC​CCC​TCA​AG. β-actin-F: CTTCGCGGGCGACGAT, β-actin-R: CCA​TAG​GAA​TCC​TTC​TGA​CC. The expression quantification was obtained with the 2^−ΔΔCT^ method (ns, *p* ≥ 0.05; *, *p* < 0.05; **, *p* < 0.01; ***, *p* < 0.001).

### Cell Proliferation and Colony Formation Assays

Cell proliferation, colony formation, and tumor sphere formation assay were performed as previously documented (Xiong et al., 2021). Briefly, for cell proliferation assay, indicated cells were plated into 12-well plates at a density of 1.5 × 10^4^, and the cell numbers were subsequently counted each day using an automatic cell analyzer countstar (Shanghai Ruiyu Biotech Co., China, IC 1000). For the colony formation assay, indicated cells were seeded in a 6-well plate (China, NEST, Cat. 703001) with 600 cells per well supplemented with 2 ml cell culture medium, and the cell culture medium was changed every 3 days for 2–3 weeks, and then indicated cells were fixed with 4% PFA and stained with 0.5% crystal violet (ns, *p* ≥ 0.05; *, *p* < 0.05; **, *p* < 0.01; ***, *p* < 0.001).

### Cell Migration and Invasion Assays

Cell migration assays was performed as previously documented (Xiong et al., 2021). Briefly, to produce a wound, the monolayer cells in a 6-well plate were scraped in a straight line with pipette tips. The plate was then washed with PBS to remove detached cells. Photographs of the scratch were taken at indicated time points using Nikon inverted microscope (Ti-S) (ns, *p* ≥ 0.05; *, *p* < 0.05; **, *p* < 0.01; ***, *p* < 0.001).

### Statistical Analysis

Analysis the PAFAH1B3 expression pan-cancer was estimated using t-tests. The correlations between clinicopathological characteristics and PAFAH1B3 expression were evaluated using the Chi-squared test, Fisher exact test, Kruskal–Wallis (KW) test, Wilcoxon signed-rank test, Wilcoxon rank sum test, and logistic regression. Through univariate and multivariate analysis combined with Cox logistic regression models, other clinical factors impacting the survival and the PAFAH1B3 expression level were found. Kaplan-Meier analysis was employed to examine the survival time of patients stratified according to high or low level of the PAFAH1B3 expression. For all figures, *, **, *** indicate *p* < 0.05, *p* < 0.01, and *p* < 0.001, respectively.

## Results

### Analysis of the Expression and Prognosis Values of PAFAH1B3 in Pan-Cancer

**FIGURE 1:**
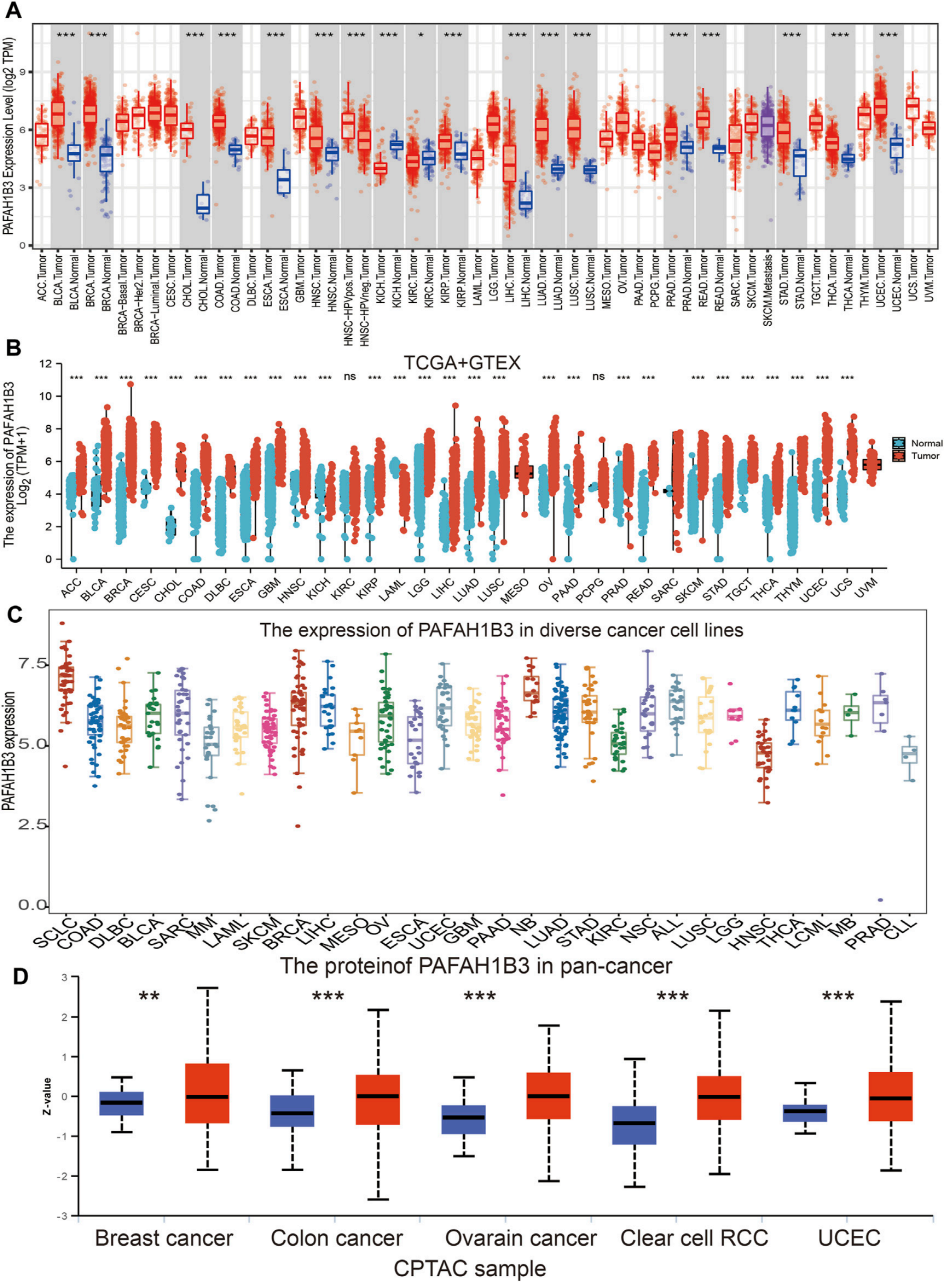
Expression analysis for PAFAH1B3 in human cancers. **(A)** The expression of PAFAH1B3 in pan-cancer analysis by TIMER database. **(B)** The expression of PAFAH1B3 in pan-cancer analysis by TCGA/GTEx database. **(C)** The expression of PAFAH1B3 in diverse cancer cells lines examine by the CCLE database. **(D)** The protein expression of PAFAH1B3 in various cancers analysis by Ualcan database.

Tumor Immune Estimation Resource (TIMER) database analysis was used to assess PAFAH1B3 in diverse cancers, and was shown to be significantly elevated in adenoid cystic carcinoma (ACC), bladder urothelial carcinoma (BLCA), cholangiocarcinoma (CHOL), colon adenocarcinoma (COAD), esophageal carcinoma (ESCA), head and neck squamous cell carcinoma (HNSC), kidney renal papillary cell carcinoma (KIRP), LIHC, lung adenocarcinoma (LUAD), lung squamous cell carcinoma (LUSC), prostrate adenocarcinoma (PRAD), rectum adenocarcinoma (READ), stomach adenocarcinoma (STAD), thyroid cancer (THCA), and uterine corpus endometrial carcinoma (UCEC). Interestingly, lower expression of PAFAH1B3 was observed in KICH and KIRC (Figure 1A). Given that some cancers lack normal tissue data in TCGA databases, PAFAH1B3 expression was also assessed in pan-cancers using the TCGA/GTEx databases. PAFAH1B3 expression was higher in ACC, BLCA, BRCA, cervical squamous cell carcinoma (CESC), COAD, CHOL, diffuse large B-cell lymphoma (DLBCL), ESCA, glioblastoma (GBM), HNSC, KIRP, low-grade glioma (LGG), LIHC, LUAD, LUSC, ovarian cancer (OV), pancreatic adenocarcinoma (PAAD), PRAD, READ, skin cutaneous melanoma (SKCM), STAD, tenosynovial giant cell tumor (TGCT), THCA, thymus cancer (THYM), UCEC, and uterine carcinosarcoma (UCS) (Figure 1B). In addition, Cancer Cell Line Encyclopedia (CCLE) databases analysis showed that PAFAH1B3 was overexpressed in many cancer cell lines (Figure 1C). To verify these results, UALCAN database analysis was used to assess PAFAH1B3 protein expression in human cancers. PAFAH1B3 was significantly elevated in breast cancer, colon cancer, ovarian cancer, clear cell renal cell carcinoma (RCC), and UCEC (Figure 1D). Overall, these results showed that PAFAH1B3 was upregulated in many human cancer types.

**FIGURE 2:**
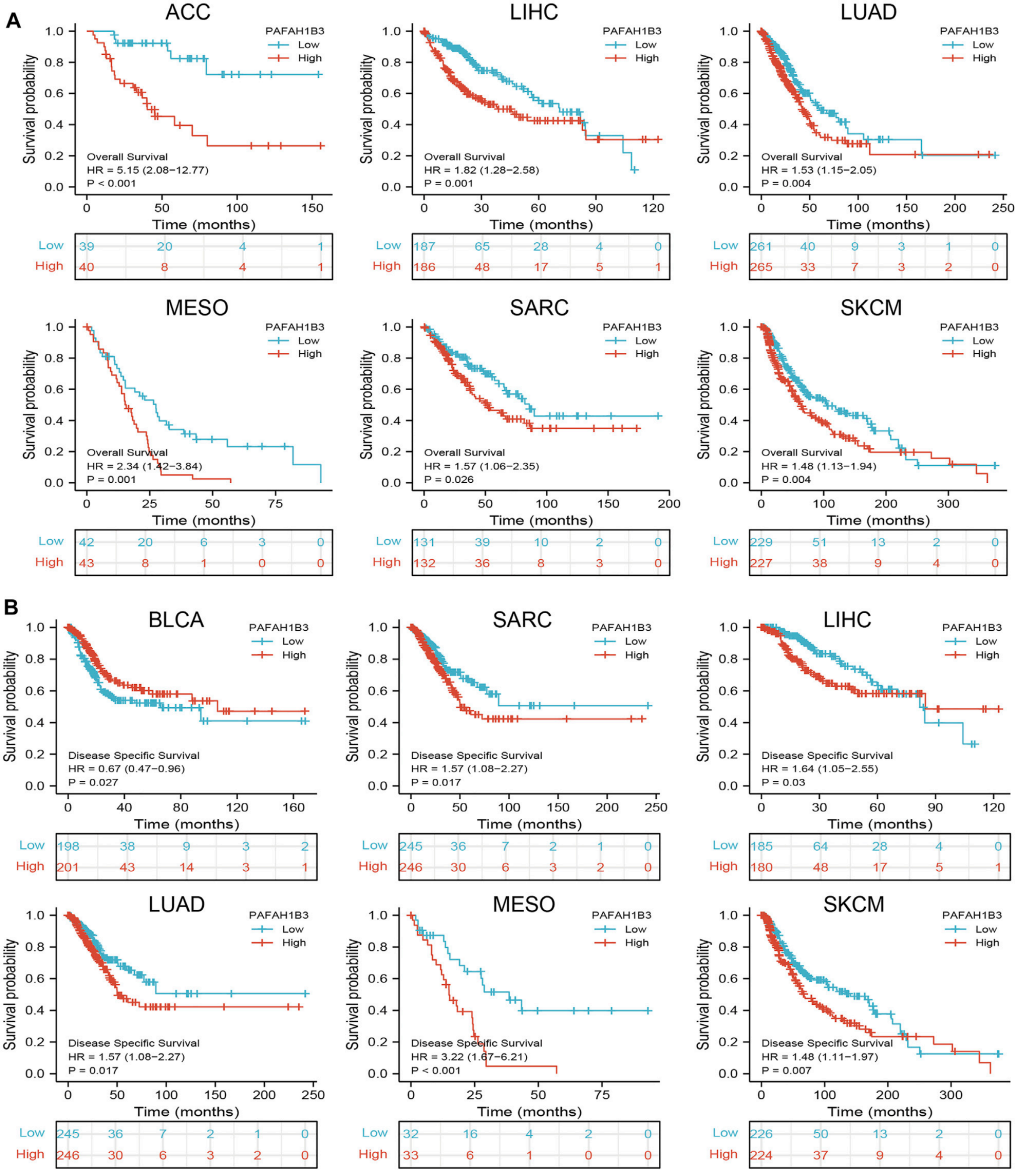
Analysis of the prognosis values for PAFAH1B3 in human cancers. **(A)** The OS of PAFAH1B3 in diverse cancers was examined by the TCGA database. **(B)** The DSS for PAFAH1B3 in pan-cancer examine by TCGA database.

Since PAFAH1B3 expression was associated with the pathology of many cancer types, the ability of PAFAH1B3 to prognose pan-cancer was explored. OS, DSS, and PFI analysis of various cancer types showed that increased PAFAH1B3 expression correlated with poor overall survival for ACC, LIHC, LUAD, mesothelioma (MESO), sarcoma (SARC), and SKCM (Figure 2A), and poor DSS in BLCA, SARC, LIHC, LUAD, MESO, and SKCM (Figure 2B).

**FIGURE 3:**
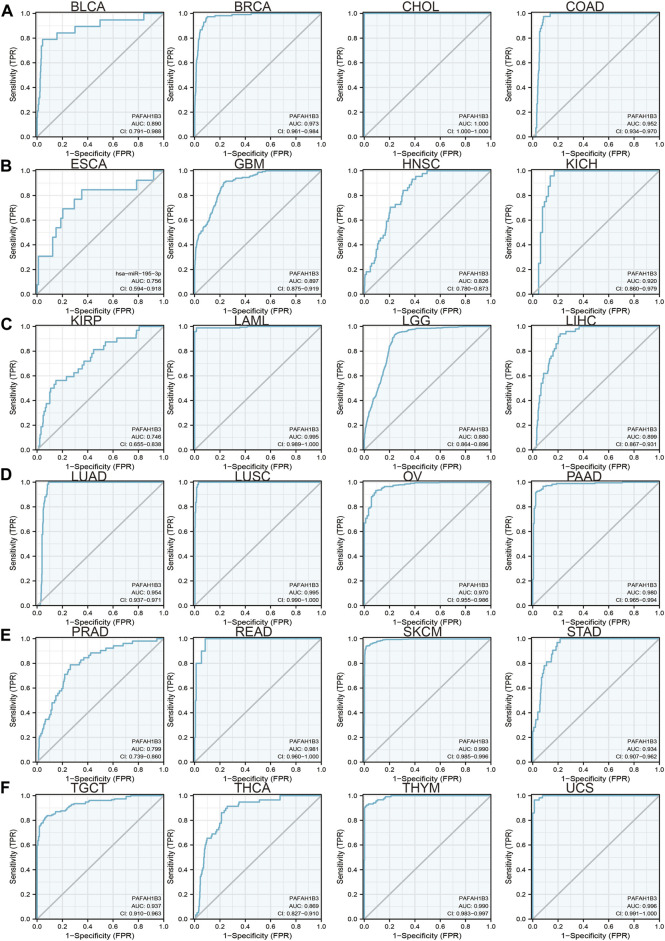
ROC curve analyses and AUC values for PAFAH1B3 in diverse cancer. **(A–D)** ROC curve analysis and AUC values for PAFAH1B3 in BLCA, BRCA, CHOL, and COAD. **(B)** ROC curve analysis and AUC values for PAFAH1B3 in ESCA, GBM, HNSC, and KICH. **(C)** ROC curve analysis and AUC values for PAFAH1B3 in KIRP, LAML, LGG, and LIHC. **(D)** ROC curve analysis and AUC values for PAFAH1B3 in LUAD, LUSC, OV, and PAAD. **(E)** ROC curve analysis and AUC values for PAFAH1B3 in PRAD, READ, STAD, and SKCM. **(F)** ROC curve analysis and AUC values for PAFAH1B3 in TGCT, THCA, THYM, and THYM.

Since previous results have shown that PAFAH1B3 expression correlates with prognosis for a wide range of cancers, this study assessed whether PAFAH1B3 may act as a detection index for cancer diagnosis. Receiver operating characteristic (ROC) curve analysis was used to assess the diagnostic value of PAFAH1B3 in various human cancers and found that it had moderate accuracy (AUC > 0.75) in predicting BLCA, BRCA, CHOL, COAD, ESCA, GBM, HNSC, KICH, KIRP, LAML, LGG, LIHC, LUAD, LUSC, OV, PAAD, PRAD, THCA, THYM, and UCS (Figures 3A–F) and high accuracy (AUC > 0.90) in predicting BRCA, CHOL, COAD, KICH, LAML, LUAD, LUSC, OV, PAAD, READ, SKCM, STAD, TGCT, THCA, THYM, and UCS. These results confirm that PAFAH1B3 has the potential to act as a detection index for the diagnosis of many cancer types with high sensitivity and specificity.

### Analysis of the Mutation Landscape of PAFAH1B3 in Various Cancer Types

**FIGURE 4:**
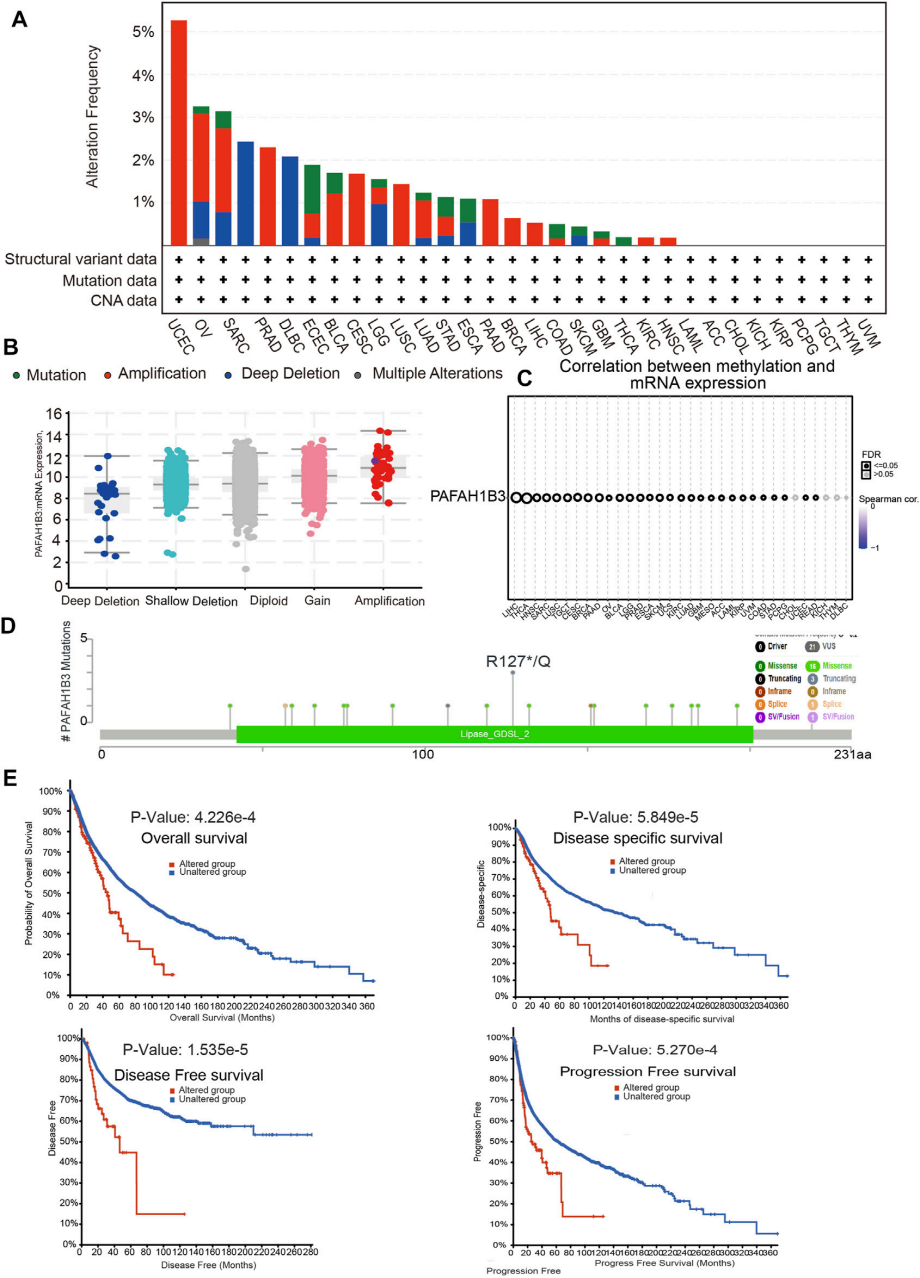
PAFAH1B3 mutation landscape in various cancer types. **(A)** PAFAH1B3 mutation level in pan-cancer examined by the cBioPortal database. **(B)** PAFAH1B3 mutation frequency in pan-cancer examined by the cBioPortal database. **(C)** The DNA methylation of PAFAH1B3 in pan-cancer **(D)** Mutation diagram of PAFAH1B3 in pan-cancer was examined by the cBioPortal database. **(E)** The correlation between PAFAH1B3 mutation and poor prognosis in patients.

Alterations in PAFAH1B3 copy numbers were assessed using the cBioPortal database. In various human cancers, the mutation frequency was higher in UCEC, OV, SARC, and PRAD than in other cancers (Figure 4A). Amplification was the most common type of alteration, followed by shallow depletion and diploid (Figure 4B). DNA methylation analysis showed that PAFAH1B3 expression was negatively associated with DNA methylation in LIHC, THCA, HNSC, and SARC (Figure 4C). To examine the PAFAH1B3 mutation landscape in various cancer types, 21 VUS, 16 missense sites, three truncation sites, one splice, and one fusion situated between amino acids 0 and 231 were identified in PAFAH1B3 using the cBioPortal database (Figure 4D). PAFAH1B3 genetic alterations were associated with overall survival, disease-specific survival, and progression-free interval in cancer patients (Figure 4E). These results confirm that PAFAH1B3 genetic alterations affect PAFAH1B3 expression and prognostic ability.

### Correlations Between PAFAH1B3 Expression and Immune and Molecular Subtypes, Tumor Mutational Burden, and Microsatellite Instability in Pan-Cancer

The correlation between PAFAH1B3 expression and development of different immune and molecular subtypes in pan-cancer was assessed using the TISIDB database. Results showed that PAFAH1B3 has different expression patterns in pan-cancer (Supplementary Figures S1A–D). For example, while PAFAH1B3 was highly expressed in C2 of LIHC, it was poorly expressed in C3. PAFAH1B3 was uniquely expressed in different molecular subtypes of cancer (Supplementary Figures S2A–D). For example, PAFAH1B3 expression was higher in the C2C-CIMP subtype of LIHC. These results confirm that PAFAH1B3 expression is associated with different immune and molecular subtypes of cancer.

### Correlations Between PAFAH1B3 Expression and Tumor Mutational Burden and Microsatellite Instability in Pan-Cancer

Tumor Mutational Burden (TMB), the number of DNA mutations in cancer, has emerged as a sensitive and specific biomarker in response to immune checkpoint inhibitors (Addeo et al., 2021). PAFAH1B3 expression was positively associated with the TMB in MESO, LUAD, STAD, PAAD, ACC, LGG, DLBC, UVM, and PRAD (*r* > 0.2, *p* < 0.01), and negatively associated with the TMB in THYM (Supplementary Figure S3A). Microsatellite instability (MSI) represents a hyper-mutable state of DNA sequences caused by the lack of DNA mismatch repair activity (Boland and Goel, 2010). PAFAH1B3 expression was positively correlated with the MSI in DLBC, STAD, PAAD, MESO, ESCA, UCEC, and LIHC (*r* > 0.15, *p* < 0.01), and negatively correlated with the MSI in TGCT, LAML, COAD, UCS, and READ (Supplementary Figure S3B).

### Correlation Between PAFAH1B3 Expression and Immune Infiltration, Drug Sensitivity

**FIGURE 5:**
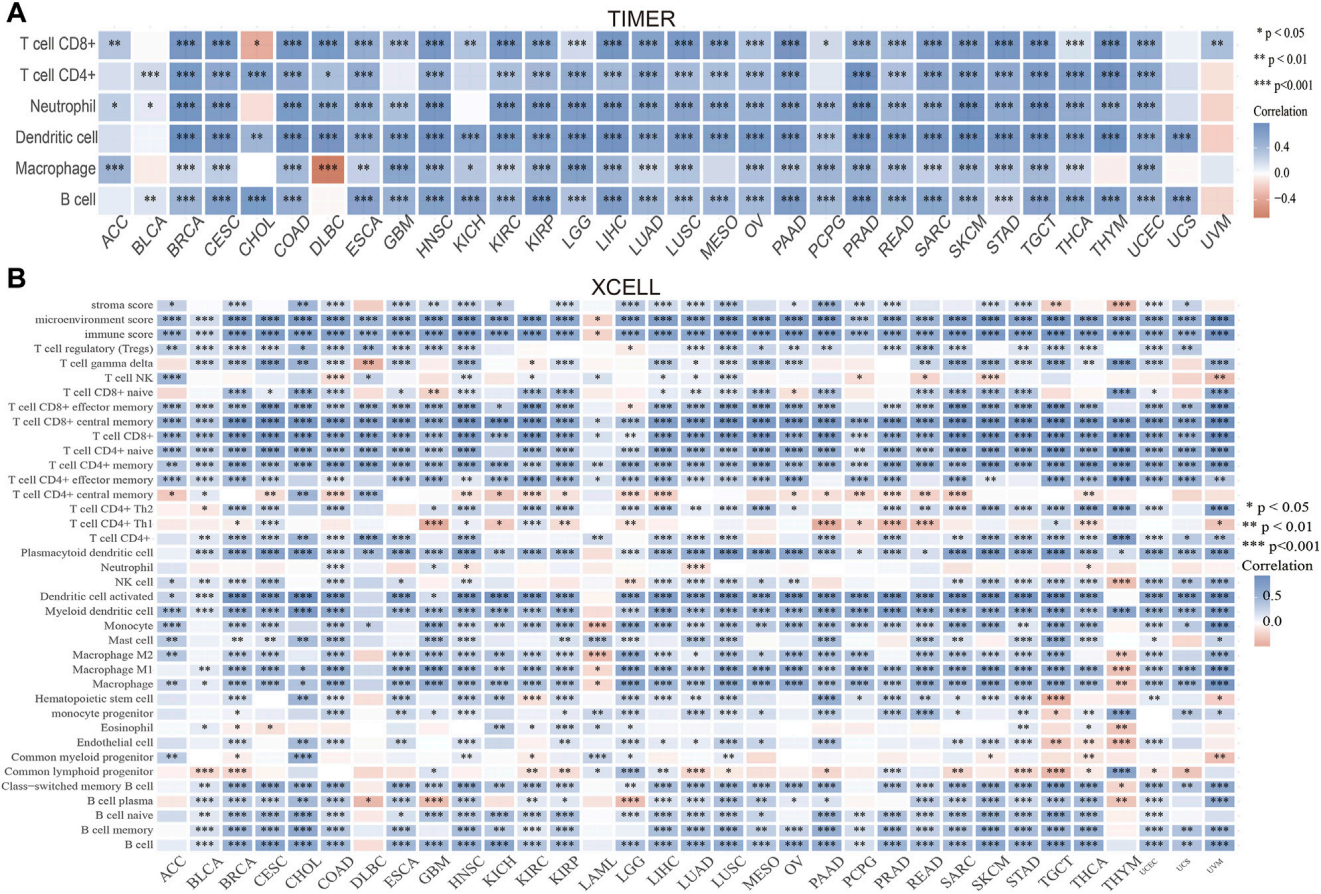
Analysis of the correlation between the PAFAH1B3 expression and immune cells infiltration. **(A)** The correlation between PAFAH1B3 expression and immune cells infiltration in pan-cancer was examined by the TIMER database. **(B)** The correlation between PAFAH1B3 expression and diverse immune cells infiltration in pan-cancer examine by Xcell database. *p < 0.05, **p < 0.01, ***p < 0.001.

Immune cell infiltration is important to cancer progression. TIMER results showed that PAFAH1B3 expression correlated with CD8^+^ T cell abundance in 27 cancers, CD4^+^ T cell abundance in 28 cancers, neutrophil abundance in 30 cancers, dendritic cell (DC) abundance in 30 cancers, macrophage abundance in 27 cancers, and B cell abundance in 29 cancers (Figure 5A). To verify these results, xCell was used to assess the correlation between PAFAH1B3 expression and immune cell infiltration in many cancer types. Expression correlated positively with 38 immune cell types in 25 cancers and correlated negatively with 32 immune cell types in two cancers (Figure 5B). Findings indicate that PAFAH1B3 expression is significantly correlated with immune cell infiltration during human cancer.

To further determine the relationship between PAFAH1B3 and the tumor microenvironment, TCGA analysis was used to measure the correlation between PAFAH1B3 and immune checkpoint-related genes. PAFAH1B3 expression was positively associated with immune checkpoint-related genes such as CD274, CTLA4, HAVCR2, LAG3, PDCD1, PDCD1LG2, SIGLEC15, and TIGIT, in 31 cancers (Supplementary Figure S4). The TISIDB tool showed that PAFAH1B3 expression was positively associated with genes for 28 tumor-infiltrating lymphocytes, 45 immune-stimulators, 24 immune-inhibitors, 41 chemokines, 18 receptors, and 21 MHCs in pan-cancer (Supplementary Figures S5A,B). These findings indicate that PAFAH1B3 plays an important role in regulating the immune response to human cancer.

The correlation between PAFAH1B3 expression and sensitivity to different drugs was assessed using cancer cell lines from the Genomics of Drug Sensitivity in Cancer (GDSC) database and the Cancer Therapeutics Response Portal (CTRP) database. In the GDSC database, PAFAH1B3 expression was positively correlated with Z-LLNle-CHO, XMD8-85, CGP-60474, A-770041, dasatinib, bortezomib, AZ628 and JW-7-52-1 (*r* > 0.17, *p* < 0.0001) sensitivity, and negatively correlated with Navitoclax, GSK1070916, WZ3105, and SB52334 (*r* < −0.10, *p* < 0.0001) sensitivity (Supplementary Figure S6A). In the CTRP database, PAFAH1B3 expression was positively correlated with trametinib, dasatinib, BRD-K44224150, and BRD-A05715709 (*r* > 0.10, *p* < 0.0001) sensitivity, and negatively associated with GSK-J4, SR8278, BRD-K48334597, MI-2, HBX-41108, pifithrin-mu, cerulenin, SB-525334, valdecoxib, belinostat, PX-12, and skepinone (*r* < −0.20, *p* < 0.0001) sensitivity (Supplementary Figure S6B). These results suggest that PAFAH1B3 is significantly associated with diverse drug sensitivity in different cancer cell lines and may be a promising therapeutic target for cancer.

### Correlation Between PAFAH1B3 and Clinical Characteristics in NSCLC and LIHC

**FIGURE 6:**
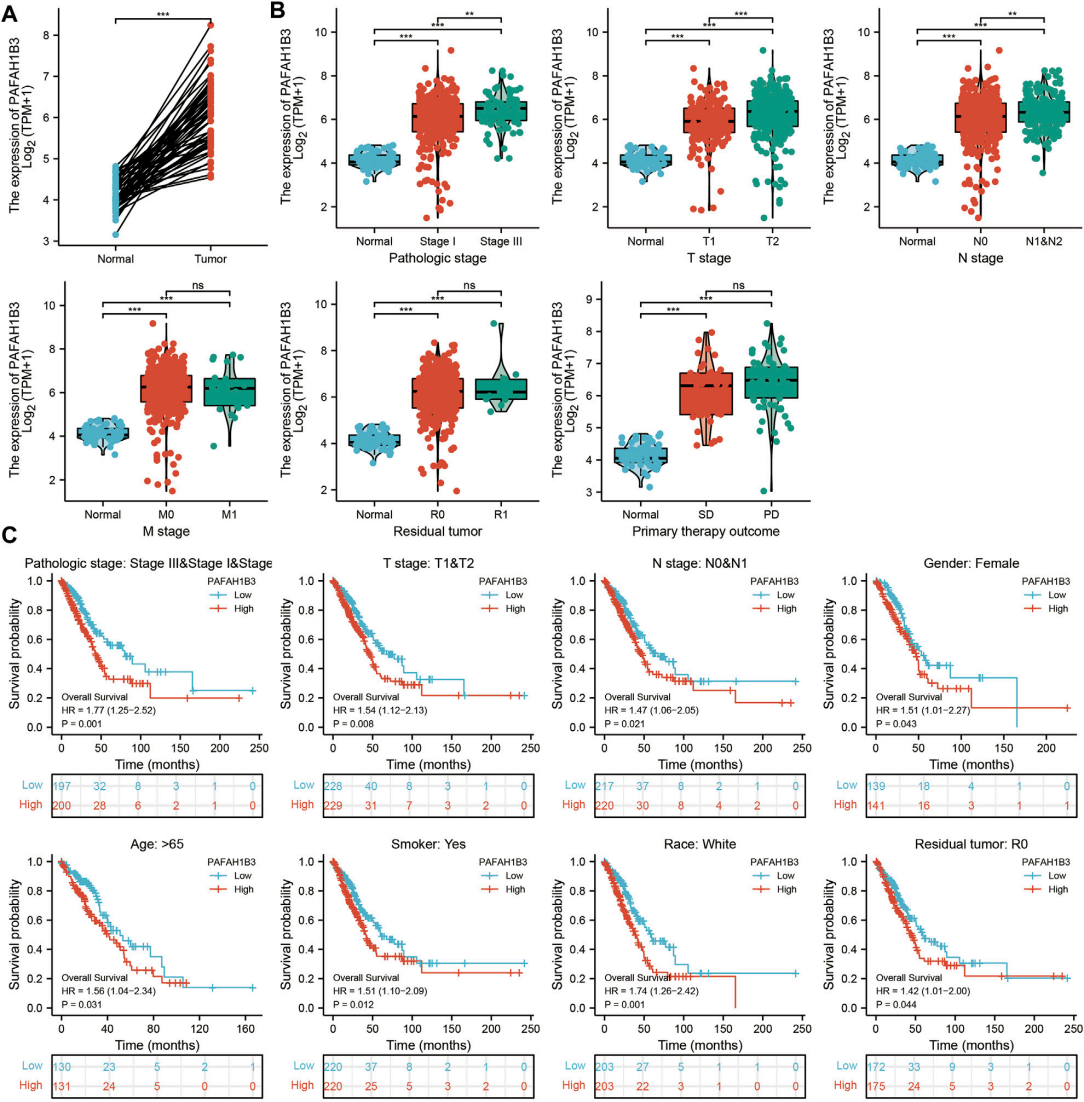
PAFAH1B3 Is Correlated With Clinical Characteristics and Prognosis in NSCLC. **(A)** The expression of PAFAH1B3 in NSCLC. **(B)** The correlation between PAFAH1B3 expression and Clinical Characteristics in NSCLC, including the pathologic stage, TNM stage, residual tumor, and primary therapy outcome. **(C)** The prognosis of PAFAH1B3 in NSCLC.

Comprehensive bioinformatics was performed to assess the correlation between PAFAH1B3 overexpression and NSCLC pathology. Overexpressed PAFAH1B3 in NSCLC (Figure 6A) was strongly associated with pathologic stage, TNM stage, residual tumor, and outcome of primary therapy (Figure 6B). High expression of PAFAH1B3 had a worse OS for most clinical and demographic NSCLC subgroups including pathologic stage, TN stage, residual tumor, gender, age, smoking status, and race (Figure 6C). These results confirm that PAFAH1B3 plays a critical role in the progression of NSCLC.

**FIGURE 7:**
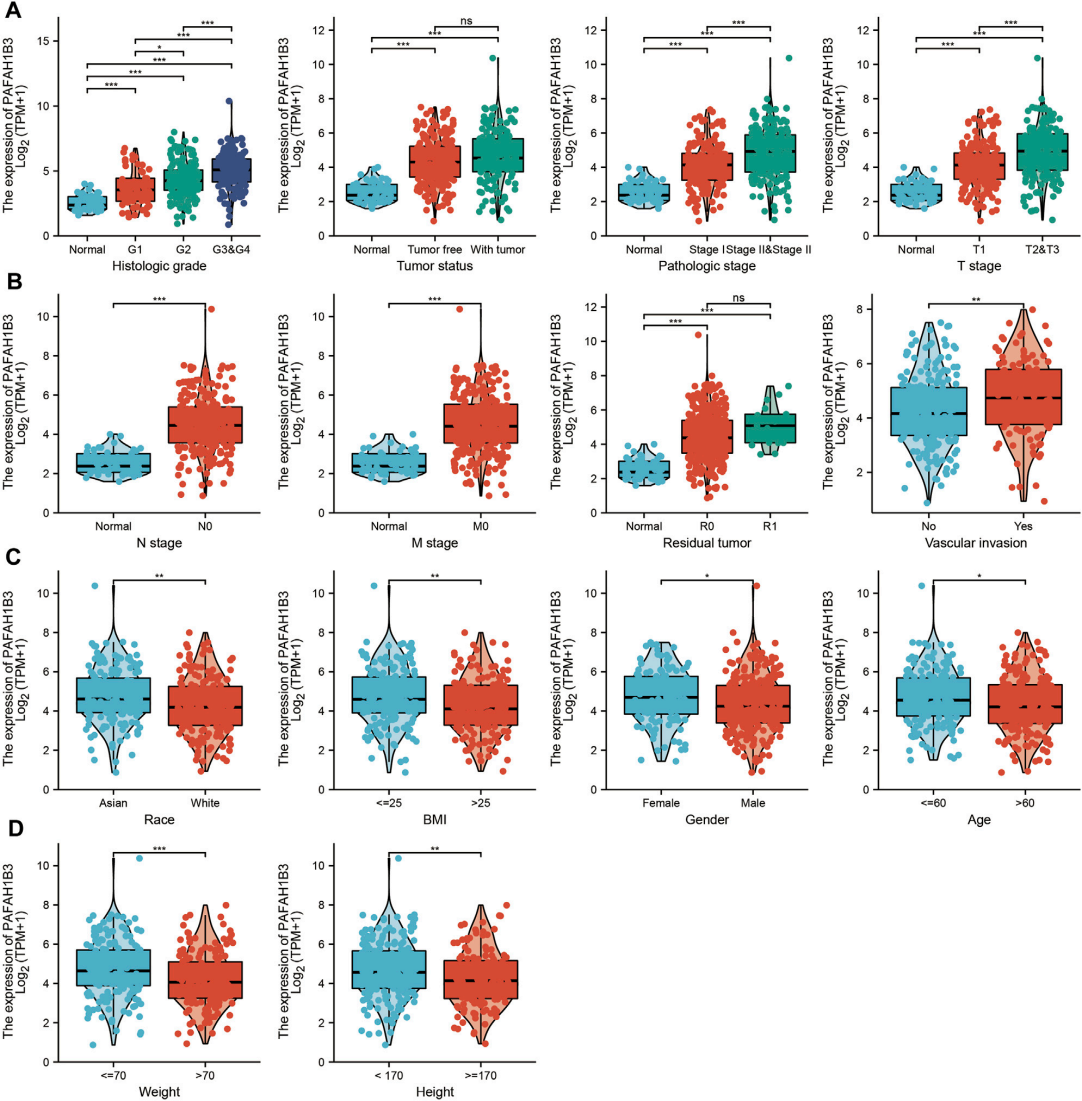
PAFAH1B3 Is Correlated With Clinical Characteristics in LIHC. **(A–D)** The correlation between PAFAH1B3 expression and Clinical Characteristics in LIHC, including the histologic stage, Tumor status, pathologic stage, TNM stage, residual tumor, vascular invasion, race, BMI, Gender, age, weight, and height.

**FIGURE 8:**
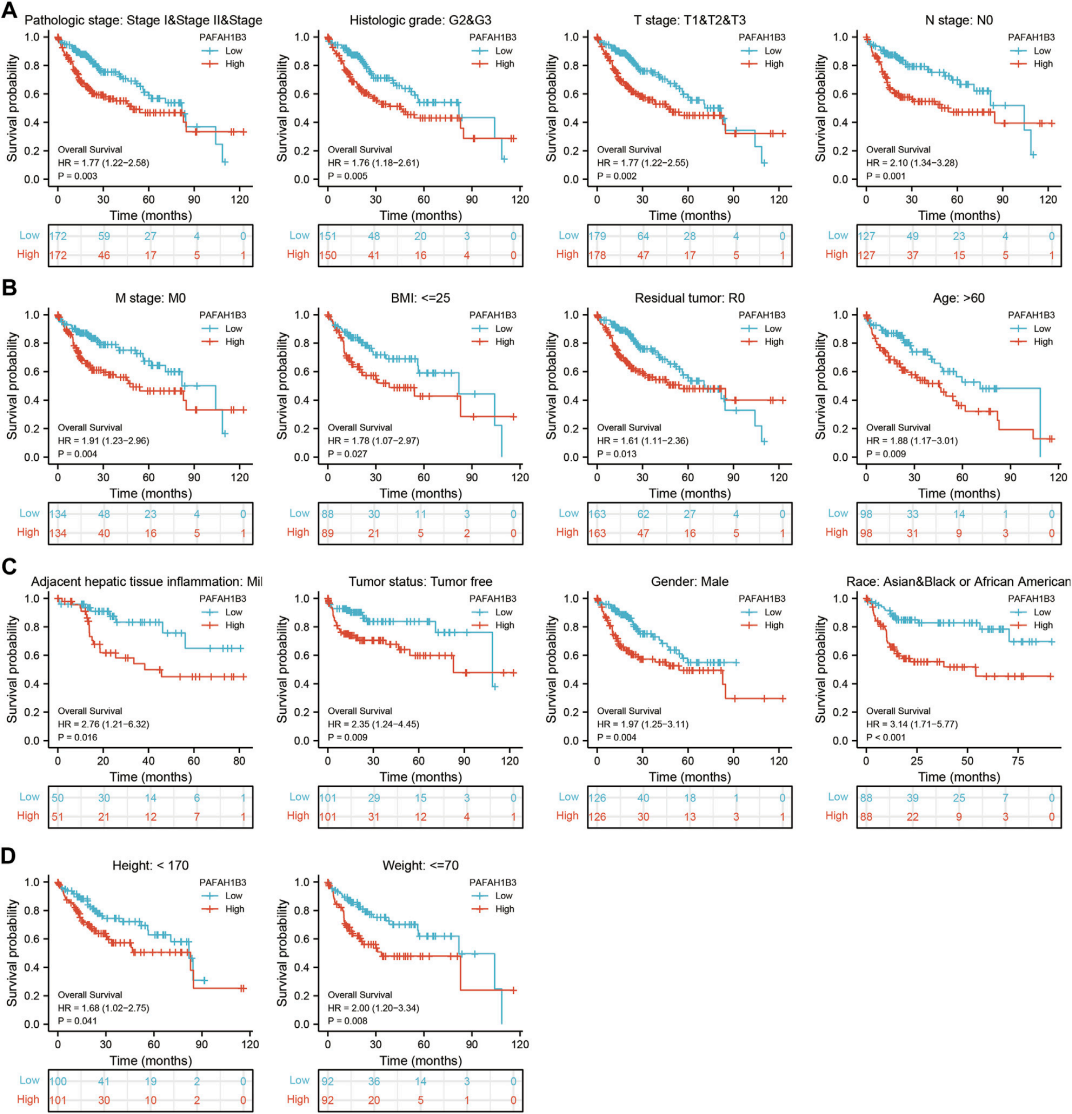
PAFAH1B3 Is Correlated With Prognosis in LIHC. **(A–D)** The correlation between PAFAH1B3 and OS in different clinical subgroups of LIHC, including pathologic stage, histologic stage, TNM stage, residual tumor, gender, age, adjacent hepatic tissue inflammation, height, and weight.

The correlation between PAFAH1B3 expression and pathology of LIHC was also assessed. High PAFAH1B3 expression was significantly associated with histologic stage, tumor status, pathologic stage, TNM stage, residual tumor, vascular invasion, race, BMI, gender, age, weight, and height (Figures 7A–D). In addition, overexpression of PAFAH1B3 had a worse OS in most clinical and demographic subgroups of LIHC, including pathologic stage, histologic stage, TNM stage, residual tumor, gender, age, adjacent hepatic tissue inflammation, height, and weight (Figures 8A–D and Table 1). Univariate and multivariate Cox regression analyses showed that TM stage, pathologic stage, tumor status, and PAFAH1B3 expression were significantly associated with OS (Table 2). These results confirm that PAFAH1B3 plays a critical role in LIHC progression.

**TABLE 1:** PAFAH1B3 expression associated with clinical pathological characteristics in LIHC (logistic regression).

Characteristics	Total(N)	Odds ratio (OR)	*p* Value
T stage (T2 and T3 vs. T1)	358	2.426 (1.591−3.723)	<0.001
Pathologic stage (Stage III & Stage IV vs. Stage I and Stage II)	350	1.922 (1.182−3.162)	0.009
Histologic grade (G3 and G4 vs. G1 & G2)	369	3.315 (2.132−5.217)	<0.001
Age (>60 vs. ≤60)	373	0.657 (0.436−0.987)	0.044
Weight (>70 vs. ≤70)	346	0.504 (0.327−0.772)	0.002
Height (≥170 vs. <170)	341	0.494 (0.317−0.764)	0.002
Race (Black or African American vs. Asian)	177	0.810 (0.295−2.262)	0.681

**TABLE 2:** Univariate and multivariate Cox regression analyses of clinical characteristics associated with OS of LIHC.

Characteristics	Total(N)	Univariate analysis		Multivariate analysis	
		Hazard ratio (95% CI)	*p* Value	Hazard ratio (95% CI)	*p* Value
T stage	370				
T1	183	Reference			
T2	94	1.428 (0.901−2.264)	0.129	1.390 (0.755−2.561)	0.291
T3 and T4	93	2.949 (1.982−4.386)	<0.001	1.776 (0.238−13.258)	0.575
M stage	272				
M0	268	Reference			
M1	4	4.077 (1.281−12.973)	0.017	1.110 (0.263−4.688)	0.887
Pathologic stage	349				
Stage I and Stage II	259	Reference			
Stage III and Stage IV	90	2.504 (1.727−3.631)	<0.001	1.483 (0.203−10.856)	0.698
Tumor status	354				
Tumor free	202	Reference			
With tumor	152	2.317 (1.590−3.376)	<0.001	1.915 (1.202−3.052)	0.006
PAFAH1B3	373	1.235 (1.095−1.392)	<0.001	1.198 (1.036−1.385)	0.015

**FIGURE 9:**
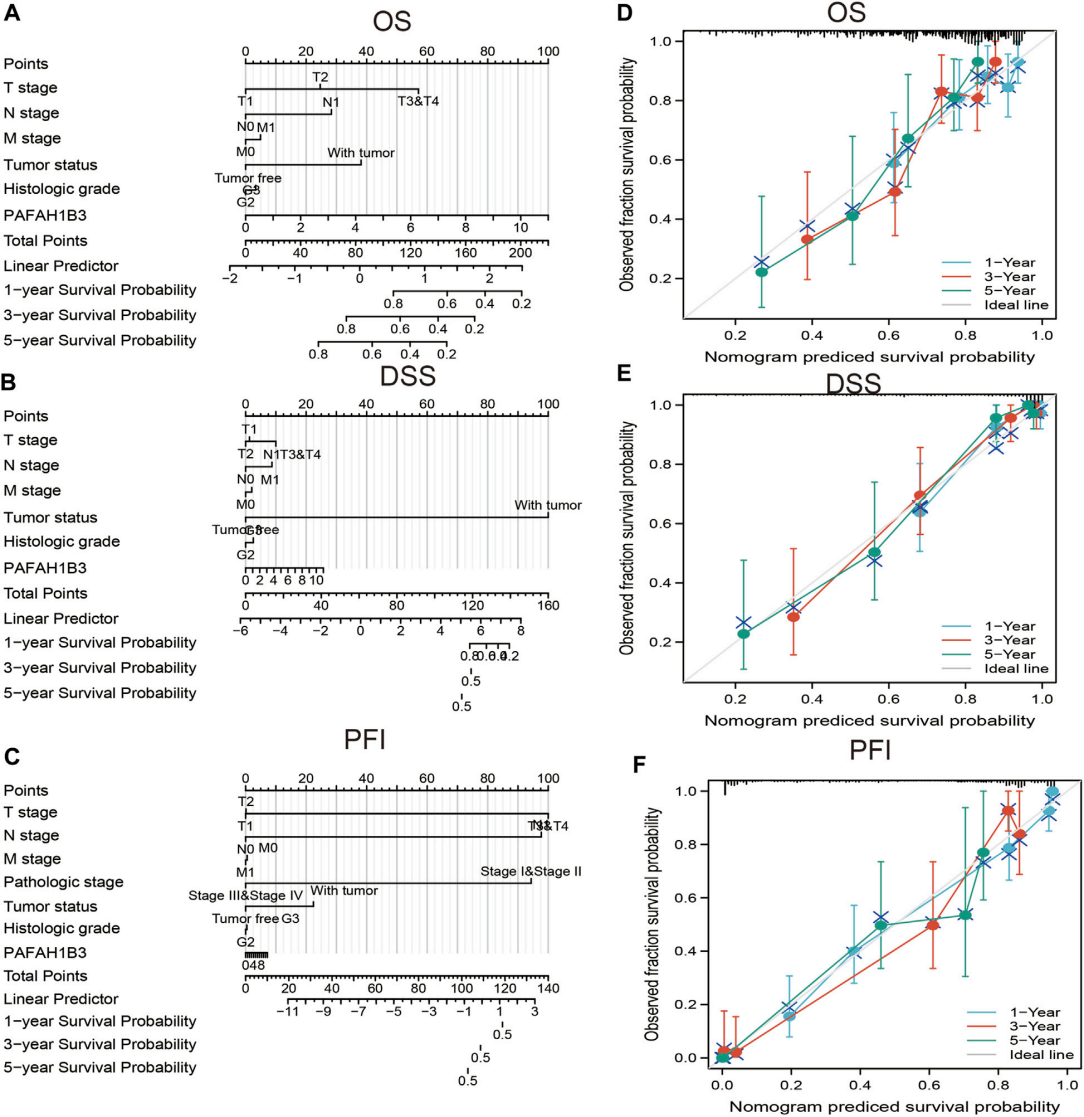
Nomogram and calibration curve for predicting the probability of 1-, 3-, and 5-years OS for LIHC patients. **(A–C)** A nomogram integrates PAFAH1B3 and other prognostic factors in LIHC from TCGA data, **(D–F)** The calibration curve of the nomogram.

A nomogram was created to integrate PAFAH1B3 as a LIHC biomarker, including the TNM stages, tumor status, histologic stage to predict OS, DSS, and PFI. The C-indices of OS, DSS, and PFI were 0.680, 0.871, and 0.808, respectively. The calibration curves all presented desirable predictions for the three nomograms for 1-, 3-, and 5-years clinical outcomes (Figures 9A–F). Thus, this nomogram may be a model for predicting LIHC survival with PAFAH1B3 than an individual prognostic factor.

### Analysis of the Biological Functions of PAFAH1B3 in LIHC

**FIGURE 10:**
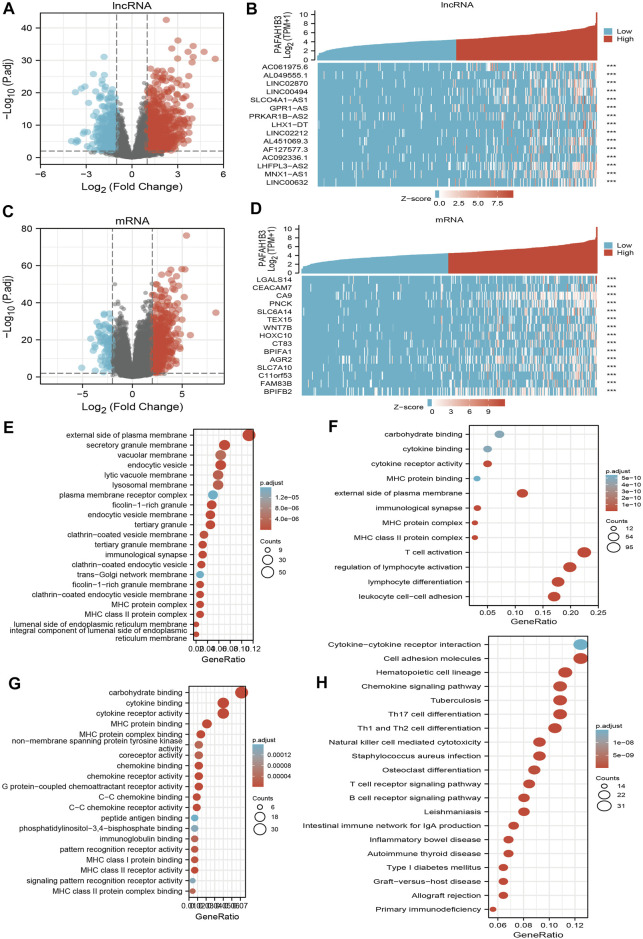
Identifying differentially expressed genes between high and low expression of PAFAH1B3 groups. **(A)** Volcano plot of differential lncRNA profiles between PAFAH1B3 high expression and PAFAH1B3 low expression. **(B)** Hot map of the top 15 DEGs (lncRNA) between PAFAH1B3 high expression and PAFAH1B3 low expression. **(C)** Volcano plot of differential mRNA profiles between PAFAH1B3 high expression and PAFAH1B3 low expression. **(D)** Hot map of the top 15 DEGs (mRNA) between PAFAH1B3 high expression and PAFAH1B3 low expression. **(E–G)** The GO term of PAFAH1B3 analysis by using differentially expressed genes. **(H)** The KEGG term of PAFAH1B3 analysis by using differentially expressed genes.

To perform Gene Ontology (GO) and KEGG enrichment analysis, 1,013 DEGs (lncRNA and mRNA) were acquired with threshold values of |log2 fold-change (FC)| > 2.0 and adjusted *p*-value < 0.01, including 813 upregulated genes and 200 downregulated genes (Figures 10A–D). For the cellular components of GO term, the DEGs mainly involved those on the external side of the plasma membrane, including MHC class II protein complex, immunological synapse, MHC protein complex, secretory granule membrane, clathrin-coated endocytic vesicle membrane, endocytic vesicle, tertiary granule membrane, tertiary granule, clathrin-coated endocytic vesicle, endocytic vesicle membrane, ficolin-1-rich granule, an integral component of the luminal side of endoplasmic reticulum membrane, luminal side of the endoplasmic reticulum membrane, colin-1-rich granule membrane, clathrin-coated vesicle membrane, and lysosomal membrane (Figure 10E). For the biology process of GO term, the DEGs mainly involved T cell activation, lymphocyte differentiation, regulation of lymphocyte activation, leukocyte cell-cell adhesion, regulation of T cell activation, regulation of leukocyte cell-cell adhesion, positive regulation of cell activation, T cell differentiation, positive regulation of leukocyte cell-cell adhesion, positive regulation of leukocyte activation, regulation of lymphocyte proliferation, regulation of mononuclear cell proliferation, regulation of leukocyte proliferation, and positive regulation of T cell activation (Figure 10F). For the molecular function of GO term, the DEGs mainly involved cytokine receptor activity, carbohydrate-binding, cytokine binding, MHC protein binding, MHC protein complex binding, MHC class II receptor activity, G protein-coupled chemoattractant receptor activity, chemokine receptor activity, C-C chemokine receptor activity, chemokine binding, C-C chemokine binding, MHC class I protein binding, pattern recognition receptor activity, coreceptor activity, and immunoglobulin binding (Figure 10G). KEGG enrichment analysis showed that the DEGs were mainly involved in hematopoietic cell lineage, Th1 and Th2 cell differentiation, Th17 cell differentiation, cell adhesion, the intestinal immune network for IgA production, allograft rejection, *Staphylococcus aureus* infection, graft-versus-host disease, type I diabetes mellitus, *Leishmaniasis* infection, autoimmune thyroid disease, B cell receptor signaling, primary immunodeficiency, T cell receptor signaling, tuberculosis, inflammatory bowel disease, natural killer cell-mediated cytotoxicity, and chemokine signaling (Figure 10H).

**FIGURE 11:**
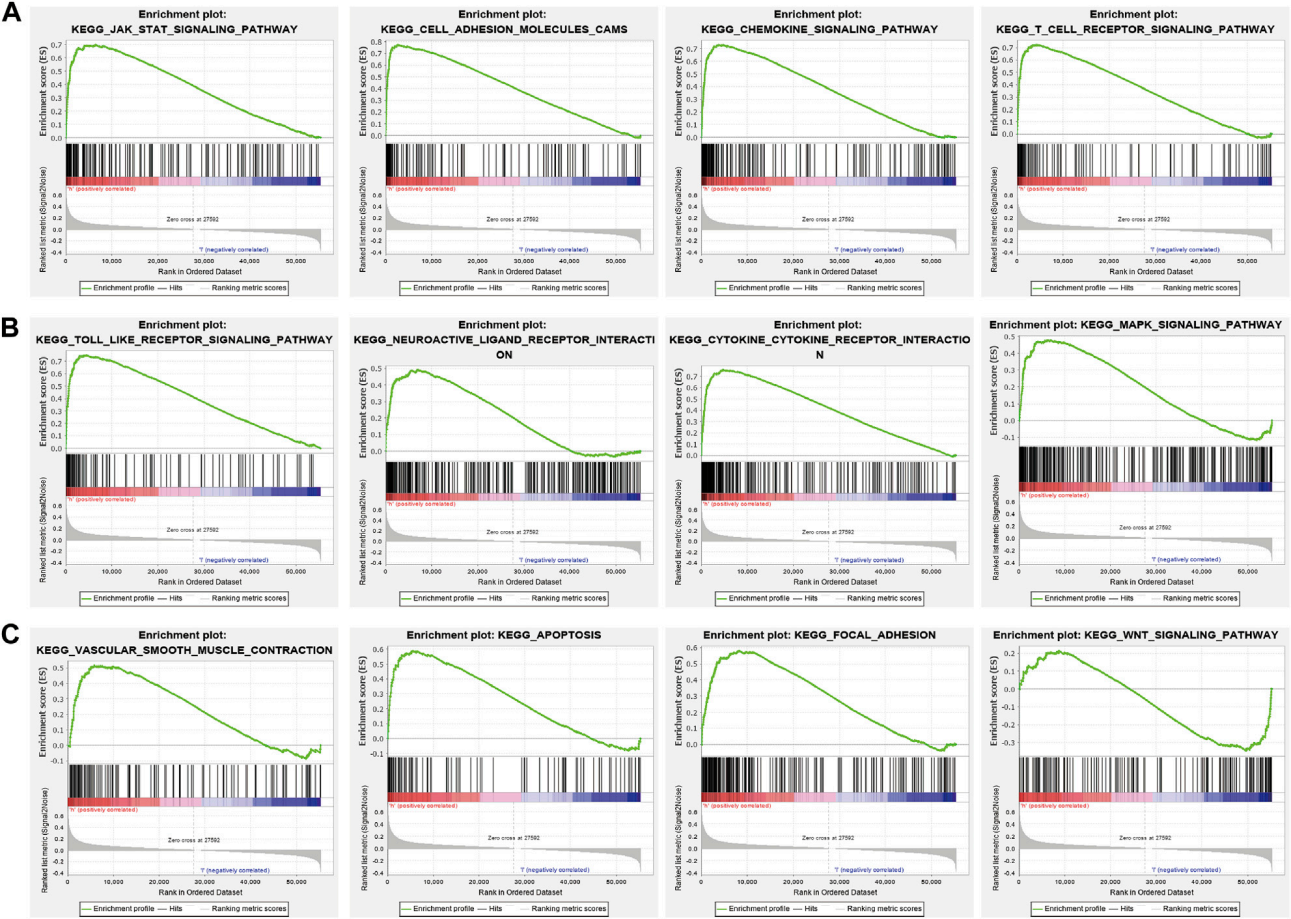
GSEA enrichment PAFAH1B3-related signaling pathway in LIHC **(A–C)** GSEA enrichment PAFAH1B3-related signaling pathway in LIHC.

GSEA enrichment of the DEGs was also assessed and results indicated that these genes mainly participated in JAK-STAT3 signaling, cell adhesion, chemokine signaling, T cell receptor signaling, Toll-like receptor signaling, neuro-active ligand-receptor interaction, cytokine receptor interaction, MAPK signaling, vascular smooth muscle contraction, apoptosis, focal adhesion, and WNT signaling (Figures 11A–C). Findings show that PAFAH1B3 plays an important role in regulating immune responses.

### PAFAH1B3 Knockdown Inhibits LIHC Cell Proliferation and Migration

**FIGURE 12:**
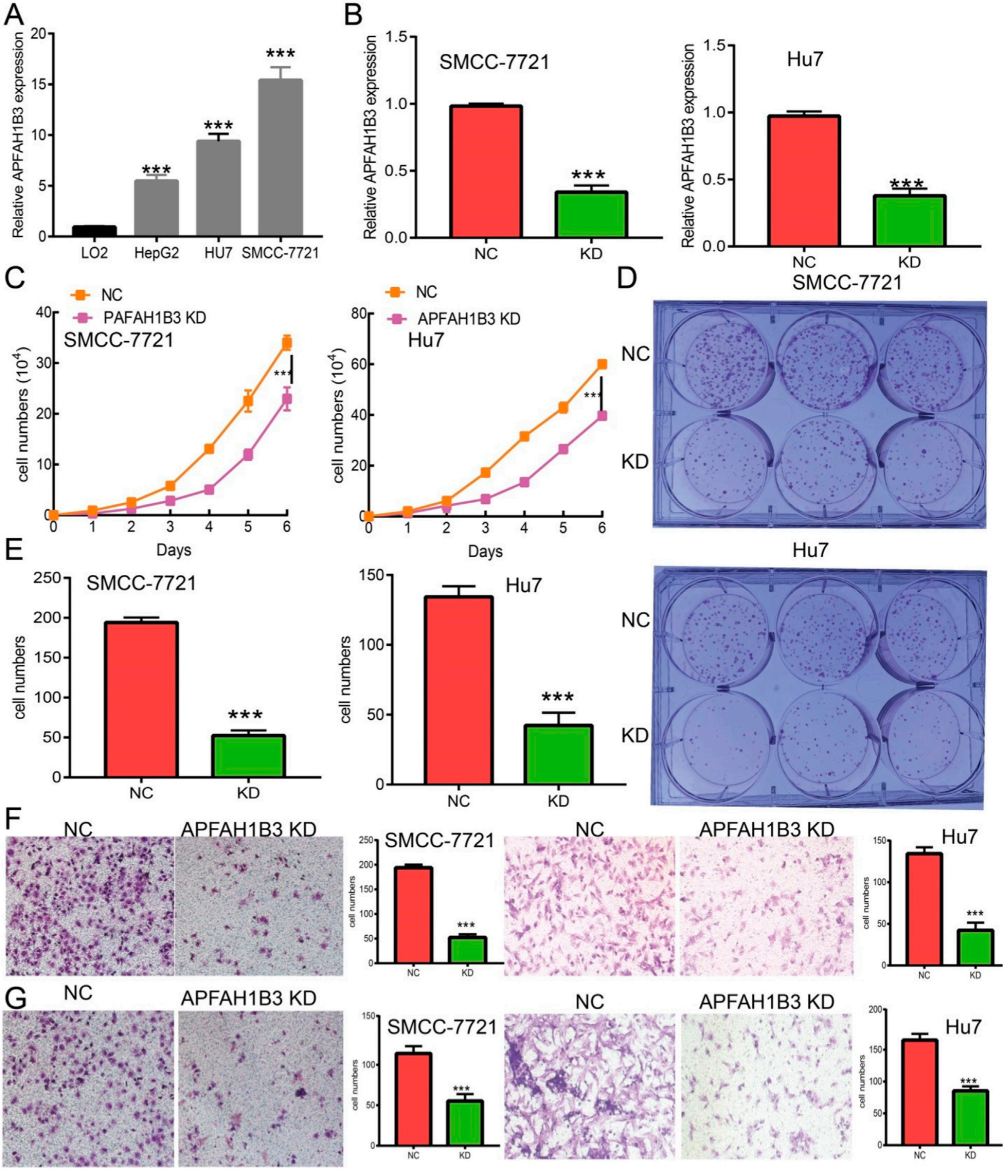
Knockdown of PAFAH1B3 inhibits LIHC progression. **(A)** The expression PAFAH1B3 in LIHC cells lines was examined by qRT-PCR assay. **(B)** Establishment of PAFAH1B3 knockdown cell lines in SMC-7721 and Hu7 verified by qRT-PCR assay. **(C)** Knockdown of PAFAH1B3 dramatically inhibits SMC-7721 and Hu7 cells proliferation examined by growth curve assay. **(D, E)** Knockdown of PAFAH1B3 dramatically inhibits SMC-7721 and Hu7 cells colony formation ability. **(F, G)** Q9 Knockdown of PAFAH1B3 dramatically inhibits SMC-7721 and Hu7 cells migration and invasion abilities.

A loss of function assay was performed to examine the functional role of PAFAH1B3 in LIHC cancer cells. PAFAH1B3 was upregulated in LIHC cell lines (Figure 12A) and inhibited by siRNA in SMC7721 and Hu7 cells. Knockdown efficacy was verified using real-time RT-PCR, using cell lines expressing a negative control as the control (Figure 12B). As expected, PAFAH1B3 knockdown inhibited SMC7721 and Hu7 cell proliferation (Figure 12C) and colony formation ability (Figures 12D,E). Trans-well and invasion assays showed that cancer cell migration and invasion were dramatically repressed in PAFAH1B3 knockdown cells compared with the control group (Figures 12F,G). These results support an oncogenic role for PAFAH1B3 in LIHC.

## Discussion

Emerging evidence shows that PAFAH1B3 plays an important role in apoptosis (Nagy et al., 2021), cancer metastasis (Monillas et al., 2015), and angiogenesis during cancer (Stafforini, 2015). Previous studies show that PAFAH1B3 is highly expressed in HSCC and is positively correlated with cervical lymph node metastasis. Depletion of PAFAH1B3 suppresses cell proliferation, migration, and induces apoptosis, thereby disrupting cell cycle processes (Xu et al., 2019). PAFAH1B3 may serve as a potential therapeutic target for HSCC patients. However, no studies have analyzed the clinical significance of PAFAH1B3 in pan-cancer.

Pan et al. found that PAFAH1B3 was more highly expressed in the HSCC tumor tissues than adjacent non-tumor samples. Moreover, increased PAFAH1B3 expression was positively correlated with cervical lymph node metastasis and adverse clinical outcome in HSCC (Xu et al., 2019). Furthermore, depletion of PAFAH1B3 inhibited the cell proliferation via modulating cell apoptosis and disrupting cell cycle process, and the migratory and invasive capacities were also attenuated in the absence of PAFAH1B3 (Xu et al., 2019). Yuan et al. found that PAFAH1B3 was overexpressed in osteosarcoma tissues and cell lines. Moreover, depletion of PAFAH1B3 inhibits the osteosarcoma cell proliferation and induced cell apoptosis *in vitro*, and also reduced osteosarcoma growth *in vivo*. This research confirmed that PAFAH1B3 could be a novel therapeutic target for osteosarcoma patients (Fan et al., 2021).

In this study, PAFAH1B3 was highly expressed in ACC, BLCA, BRCA, CESC, COAD, CHOL, DLBC, ESCA, GBM, HNSC, KIRP, LGG, LIHC, LUAD, LUSC, OV, PAAD, PRAD, READ, SKCM, STAD, TGCT, THCA, THYM, UCEC, and UCS, and high expression was associated with pathology in BRCA, HNSC, KIRC, LIHC, LUAD, TGCT, and THCA. Increased PAFAH1B3 expression also correlated with poor prognosis in ACC, LIHC, LUAD, MESO, SARC, and SKCM. Findings confirmed that PAFAH1B3 had a moderate accuracy (AUC > 0.75) in predicting BLCA, BRCA, CHOL, COAD, ESCA, GBM, HNSC, KICH, KIRP, LAML, LGG, LIHC, LUAD, LUSC, OV, PAAD, PRAD, THCA, THYM, and UCS, confirming that PAFAH1B3 has the potential to act as a detection index for the diagnosis of diverse cancers.

PAFAH1B3 expression was also associated with immune and molecular subtypes of cancer, including UCEC, BLCA, BRCA, STAD, SKCM, MESO, LUSC, LUAD, LIHC, LGG, CESC, and KIRC. TMB and MSI play a significant role in tumor immunotherapy. PAFAH1B3 expression was positively correlated with the TMB in MESO, LUAD, STAD, PAAD, ACC, LGG, DLBC, UVM, and PRAD, negatively correlated with the TMB in THYM, positively correlated with the MSI in DLBC, STAD, PAAD, MESO, ESCA, UCEC, and LIHC, and negatively correlated with the MSI in TGCT, LAML, COAD, UCS, and READ. These results confirm that PAFAH1B3 may serve as a tumor immunotherapy-related biomarker. PAFAH1B3 was primarily involved in cell proliferation and oxidative phosphorylation signaling pathways in various cancers.

Chen et al. showed that PAFAH1B3 was up-regulated in gastric cancer. High PAFAH1B3 expression was significantly correlated with high M1 macrophage and CD8-positive T cell infiltration scores. PAFAH1B3 knockdown inhibited the proliferation, migration, and the activation of oncogenic signaling in gastric cancer cells (Xie et al., 2021). Immune cell infiltration plays an indispensable role in cancer progression. In this study, PAFAH1B3 expression was significantly correlated with CD8^+^ T cell abundance in 27 cancers, CD4^+^ T cell abundance in 28 cancers, neutrophil abundance in 30 cancers, DC abundance in 30 cancers, macrophage abundance in 27 cancers, and B cell abundance in 29 cancers. PAFAH1B3 expression also correlated positively with immune checkpoint-related genes such as CD274, CTLA4, HAVCR2, LAG3, PDCD1, PDCD1LG2, SIGLEC15, and TIGIT in 31 cancers. TISIDB analysis showed that PAFAH1B3 expression was positively associated with genes for 28 tumor-infiltrating lymphocytes, 45 immune-stimulators, 24 immune-inhibitors, 41 chemokines, 18 receptors, and 21 MHCs in pan-cancer. These findings indicate that PAFAH1B3 plays an important role in regulating the immune response during human cancer. PAFAH1B3 was also significantly associated with diverse drug sensitivity in many cancer cell lines and maybe a promising therapeutic target for cancer.

This study further assessed the correlation between PAFAH1B3 and clinical characteristics and prognosis of NSCLC. High PAFAH1B3 expression was significantly associated with pathologic stage, TNM stage, residual tumor, and primary therapy outcome. Higher expression of PAFAH1B3 had a worse OS in most clinical and demographic subgroups of NSCLC, including pathologic stage, TN stage, residual tumor, gender, age, smoking status, and race.

As the most common subtype of NSCLC, accumulating evidence has confirmed that LUAD and LUSC differ from each other in their bio-pathology, molecular, clinical characteristics, and therapeutic effect (Faruki et al., 2017). In this study, we found that high PAFAH1B3 expression was significantly associated with histologic stage, tumor status, pathologic stage, TNM stage, residual tumor, vascular invasion, race, BMI, gender, age, weight, and height in LIHC. Univariate and multivariate Cox regression analyses showed that TM stage, pathologic stage, tumor status, and PAFAH1B3 expression were significantly associated with the OS. TNM stages, tumor status, histologic stage, and PAFAH1B3 expression were also included in a nomogram to predict OS, DSS, and PFI during LIHC. The C-indices of OS, DSS, and PFI were 0.680, 0.871, and 0.808, respectively.

Previous study showed that PAFAH1B3 plays a functional role in spindle formation and meiotic progression during bovine oocyte maturation (Vandenberghe et al., 2018). Aberrant higher expression of PAFAH1B3 promotes the cell proliferation and inhibits cell apoptosis of osteosarcoma (Xu et al., 2019). Recent study confirmed that high PAFAH1B3 expression was associated with high M1 macrophage and CD8-positive T cell infiltration scores (Xie et al., 2021).

To better understand the role of PAFAH1B3 in LIHC, KEGG enrichment analysis showed that these DEGs were primarily involved in the hematopoietic cell lineage, Th1 and Th2 cell differentiation, Th17 cell differentiation, cell adhesion, the intestinal immune network for IgA production, allograft rejection, *Staphylococcus aureus* infection, graft-versus-host disease, type I diabetes mellitus, *Leishmaniasis* infection, autoimmune thyroid disease, B cell receptor signaling, primary immunodeficiency, T cell receptor signaling, tuberculosis, inflammatory bowel disease, natural killer cell-mediated cytotoxicity, and chemokine signaling.

Bastian et al. found that platelet-activating factor acetylhydrolase expression in BRCA1 Mutant Ovarian cancer as a protective factor and potential negative regulator of the Wnt Signaling pathway. In this study, we showed that high PAFAH1B3 expression was associated with the JAK-STAT3 signaling, cell adhesion, chemokine signaling, T cell receptor signaling, Toll-like receptor signaling, neuro-active ligand-receptor interaction, cytokine receptor interaction, MAPK signaling, vascular smooth muscle contraction, apoptosis, focal adhesion, and Wnt signaling.

PAFAH1B3 is overexpressed in gastric cancer and knockdown of PAFAH1B3 inhibits proliferation, migration, and activation of oncogenic signaling in gastric cancer cells (Xie et al., 2021). Findings from this study showed that PAFAH1B3 was upregulated in LIHC cancer cell lines and knockdown of PAFAH1B3 inhibited the proliferation, migration, and invasion ability of LIHC cancer cells. These results demonstrate that PAFAH1B3 expression is correlated with LIHC progression.

## Conclusion

In summary, this study showed that PAFAH1B3 was elevated in multiple types of human cancer, and high expression correlated with poor prognosis. High expression of PAFAH1B3 was also associated with TMB, MSI, immune cell infiltration, and sensitivity to multiple cancer drugs. Finally, PAFAH1B3 was shown to play a critical role in the progression of LIHC, in part by promoting cell proliferation, migration, and invasion. Results indicate that PAFAH1B3 may serve as a biomarker for the clinical detection of cancer. This study provides the first evidence that PAFAH1B3 impacts cancer progression and immune responses to human pan-cancer.

## Data Availability

The datasets presented in this study can be found in online repositories. The names of the repository/repositories and accession number(s) can be found in the article/Supplementary Material.

## Glossary

ACC: Adrenocortical carcinoma
BLCA: Bladder Urothelial Carcinoma
BRCA: Breast invasive carcinoma
CESC: Cervical squamous cell carcinoma and endocervical adenocarcinoma
CHOL: Cholangiocarcinoma
COAD: Colon adenocarcinoma
DLBC: Lymphoid Neoplasm Diffuse Large B-cell Lymphoma
ESCA: Esophageal carcinoma
GBM: Glioblastoma multiforme
HNSC: Head and Neck squamous cell carcinoma
KICH: Kidney Chromophobe
KIRC: Kidney renal clear cell carcinoma
KIRP: Kidney renal papillary cell carcinoma
LAML: Acute Myeloid Leukemia
LGG: Brain Lower Grade Glioma
LIHC: Liver hepatocellular carcinoma
LUAD: Lung adenocarcinoma
LUSC: Lung squamous cell carcinoma
MESO: Mesothelioma
OV: Ovarian serous cystadenocarcinoma
PAAD: Pancreatic adenocarcinoma
PCPG: Pheochromocytoma and Paraganglioma
PRAD: Prostate adenocarcinoma
READ: Rectum adenocarcinoma
SARC: Sarcoma
SKCM: Skin Cutaneous Melanoma
STAD: Stomach adenocarcinoma
TGCT: Testicular Germ Cell Tumors
THCA: Thyroid carcinoma
THYM: Thymoma
UCEC: Uterine Corpus Endometrial Carcinoma
UCS: Uterine Carcinosarcoma
UVM: Uveal Melanoma
