# Corrigendum: Comprehensive analysis of the prognostic and immunological role of PAFAH1B in pan-cancer

**DOI:** 10.3389/fmolb.2024.1497296

**Published:** 2024-10-08

**Authors:** Yixiao Yuan, Xiulin Jiang, Lin Tang, Juan Wang, Lincan Duan

**Affiliations:** ^1^ Department of Thoracic Surgery, The Third Affiliated Hospital of Kunming Medical University, Kunming, China; ^2^ Key Laboratory of Animal Models and Human Disease Mechanisms of Chinese Academy of Sciences and Yunnan Province, Kunming Institute of Zoology, Kunming, China

**Keywords:** PAFAH1B3, pan-cancer, immune cell infiltration, drug sensitivity, prognostic biomarker, multi-omics integrative analysis, cell proliferation, cell migration

In the published article, there was an error in Figure 12 as published. The representative pictures of transwell, SMCC-7721 cells in Figure 12G were presented incorrectly. The corrected Figure 12 and its caption appear below.

**FIGURE 12:**
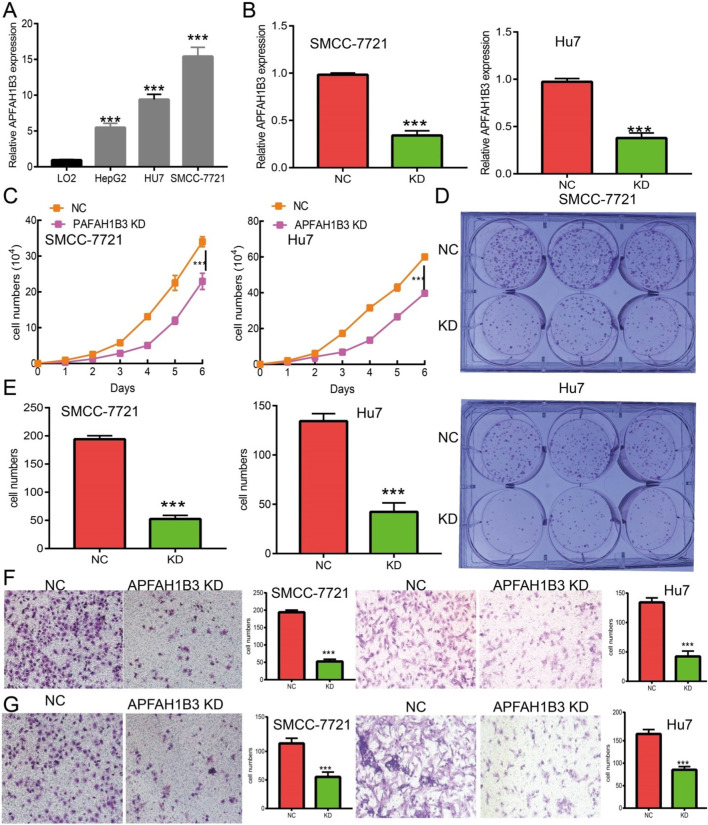
Knockdown of PAFAH1B3 inhibits LIHC progression. **(A)** The expression PAFAH1B3 in LIHC cells lines was examined by qRT-PCR assay. **(B)** Establishment of PAFAH1B3 knockdown cell lines in SMC-7721 and Hu7 verified by qRT-PCR assay. **(C)** Knockdown of PAFAH1B3 dramatically inhibits SMC-7721 and Hu7 cells proliferation examined by growth curve assay. **(D, E)** Knockdown of PAFAH1B3 dramatically inhibits SMC-7721 and Hu7 cells colony formation ability. **(F, G)** Knockdown of PAFAH1B3 dramatically inhibits SMC-7721 and Hu7 cells migration and invasion abilities.

The authors apologize for this error and state that this does not change the scientific conclusions of the article in any way. The original article has been updated.

