# Supplementary material for: Comprehensive Analysis of the Prognostic and Immunological Role of PAFAH1B in Pan-Cancer
Source: Front Mol Biosci. 2022 Feb 3;8:799497. doi: 10.3389/fmolb.2021.799497 (PMC8853080; doi:10.3389/fmolb.2021.799497)
Supplement: Supplementary file 1 [file DataSheet1.docx]

**
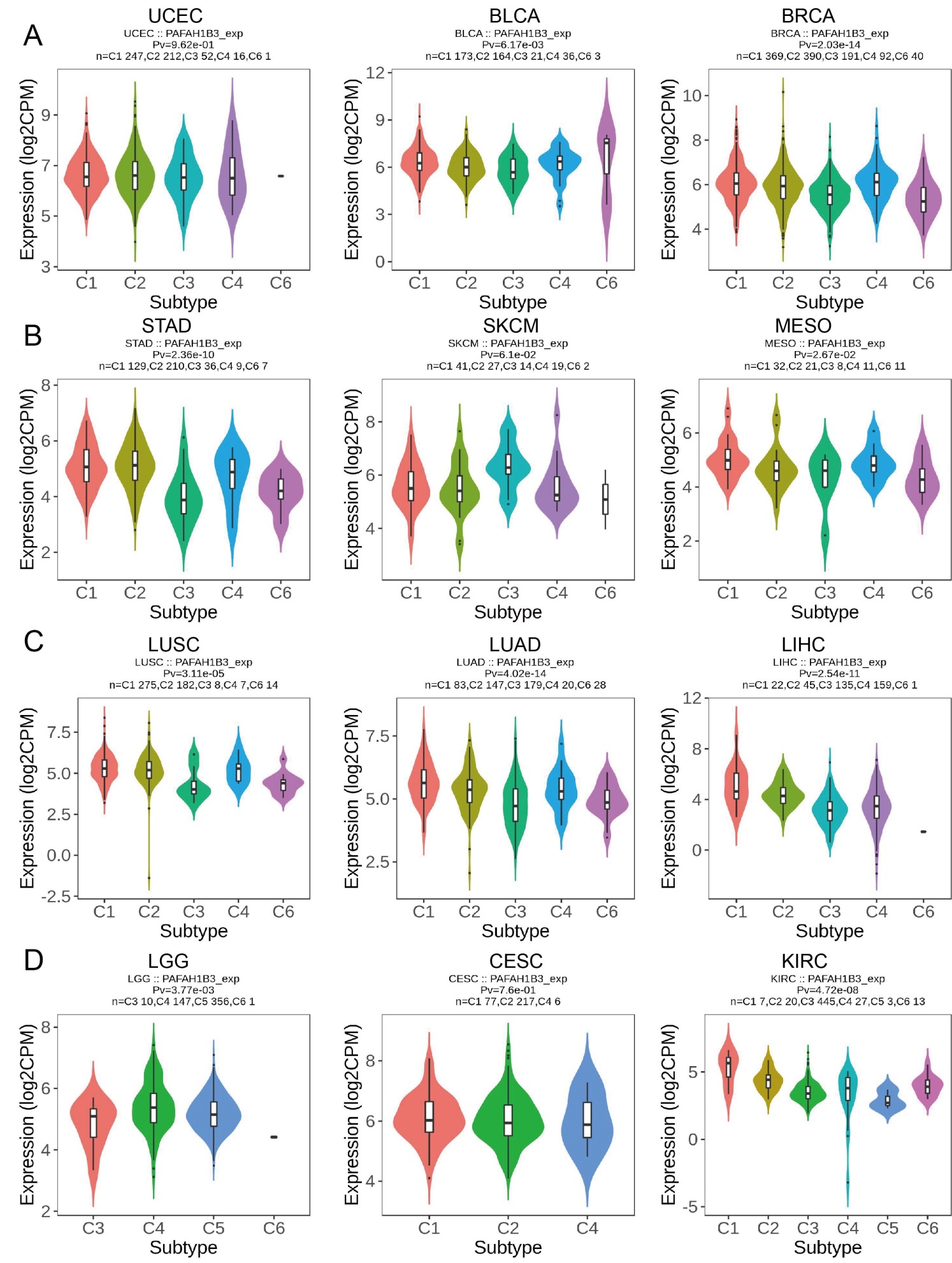
**

**Supplementary FIGURE 1⎜ The expression of PAFAH1B3 in immune subtypes of pan-cancer.**

The expression of PAFAH1B3 in UCEC, BLCA and BRCA (A),STAD, SKCM and MESO(B), LUAS, LUSC and LIHC (C) LGG, CESC and KIRC (D) immune subtypes analysis by the TISIDB database.


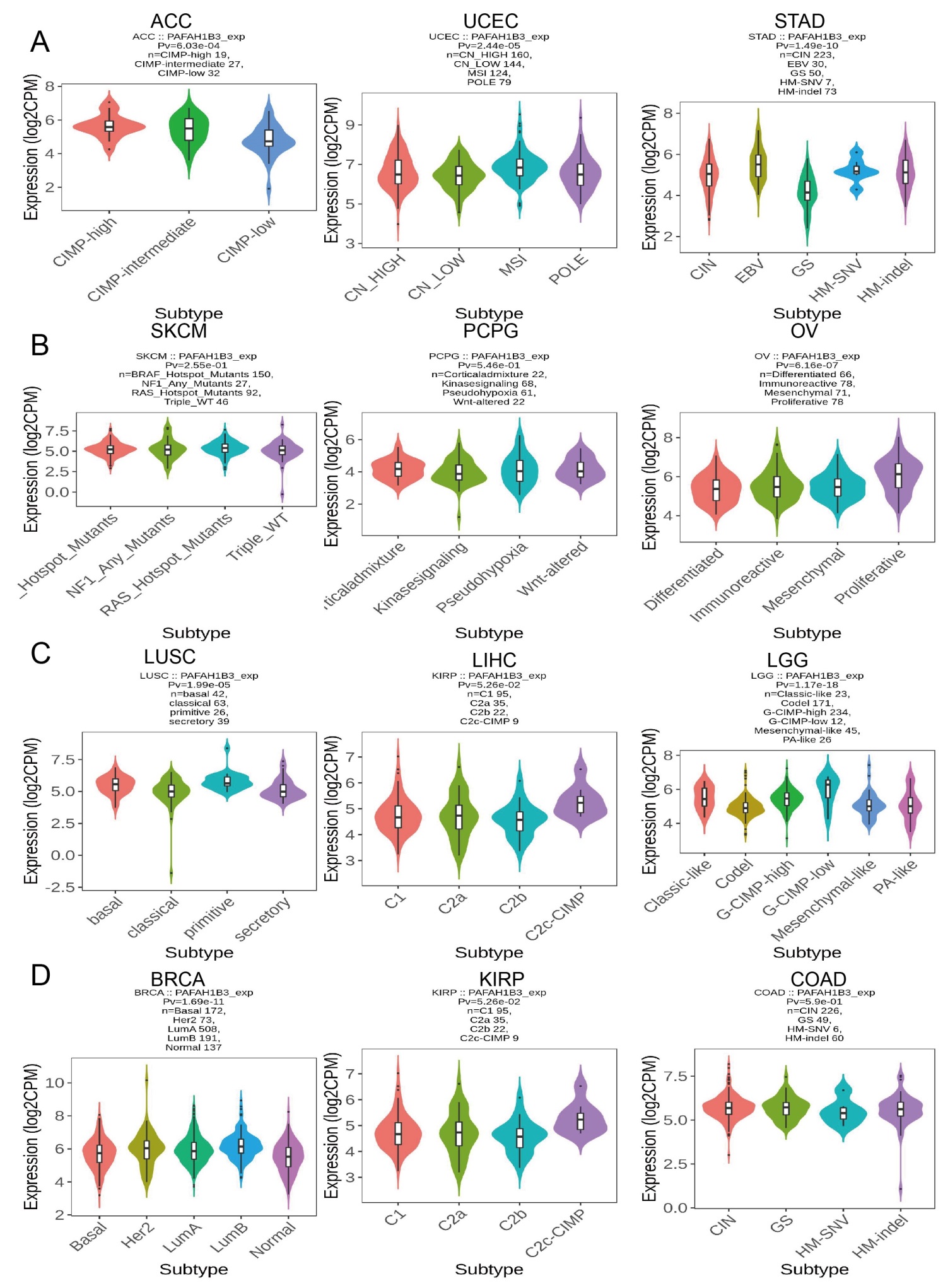


**Supplementary FIGURE 2 ⎜ The expression of PAFAH1B3 in molecular subtypes of pan-cancer.** The expression of PAFAH1B3 in ACC, UCEC and STAD (A), SKCM, PCPG and OV (B), LUSC, LIHC and LGG(C), BRCA, KIRP and COAD (D) molecular subtypes analysis by the TISIDB database.


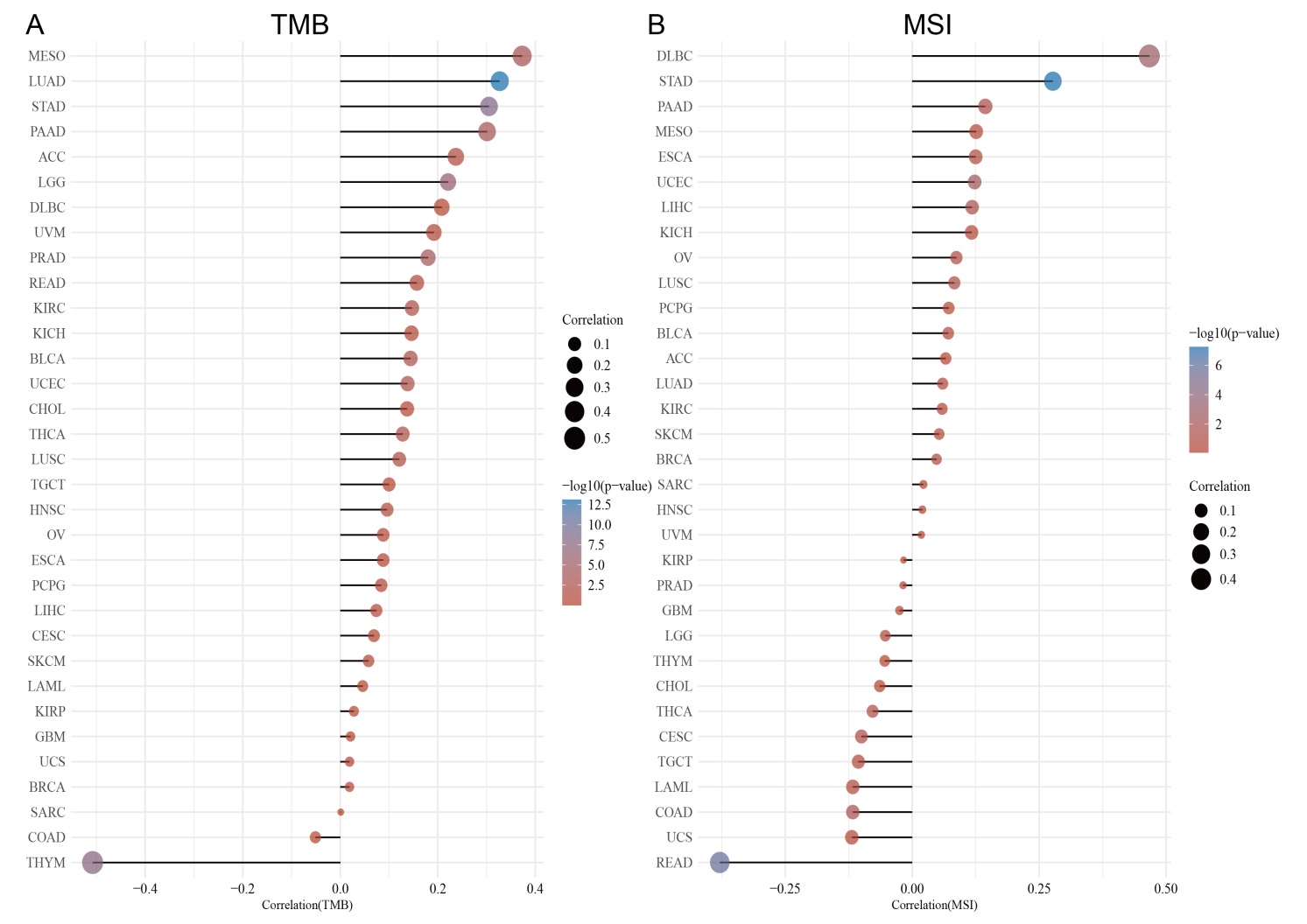


**Supplementary FIGURE 3 ⎜ The correlation between the PAFAH1B3 expression and TMB, MSI in pan-cancer.**

The correlation between the PAFAH1B3 expression and TMB (A), MSI (B).


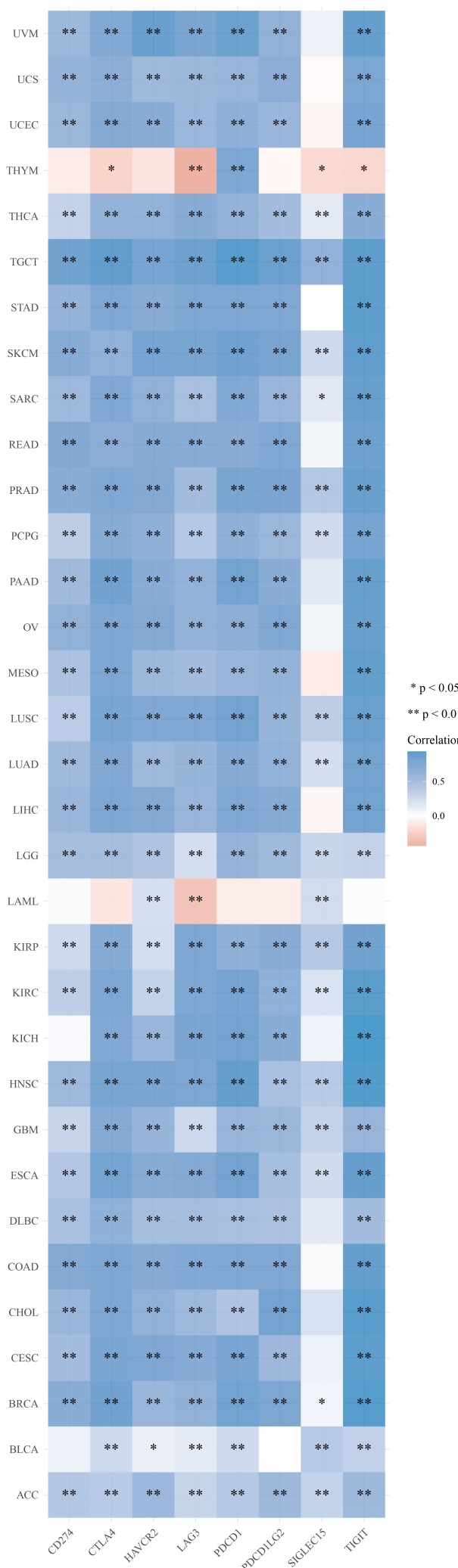


**Supplementary FIGURE 4 ⎜ Analysis the correlation between the PAFAH1B3 expression and immune check points related gene.** Analysis the correlation between the PAFAH1B3 expression and immune check points related gene in pan-cancer analysis by TIMER database.


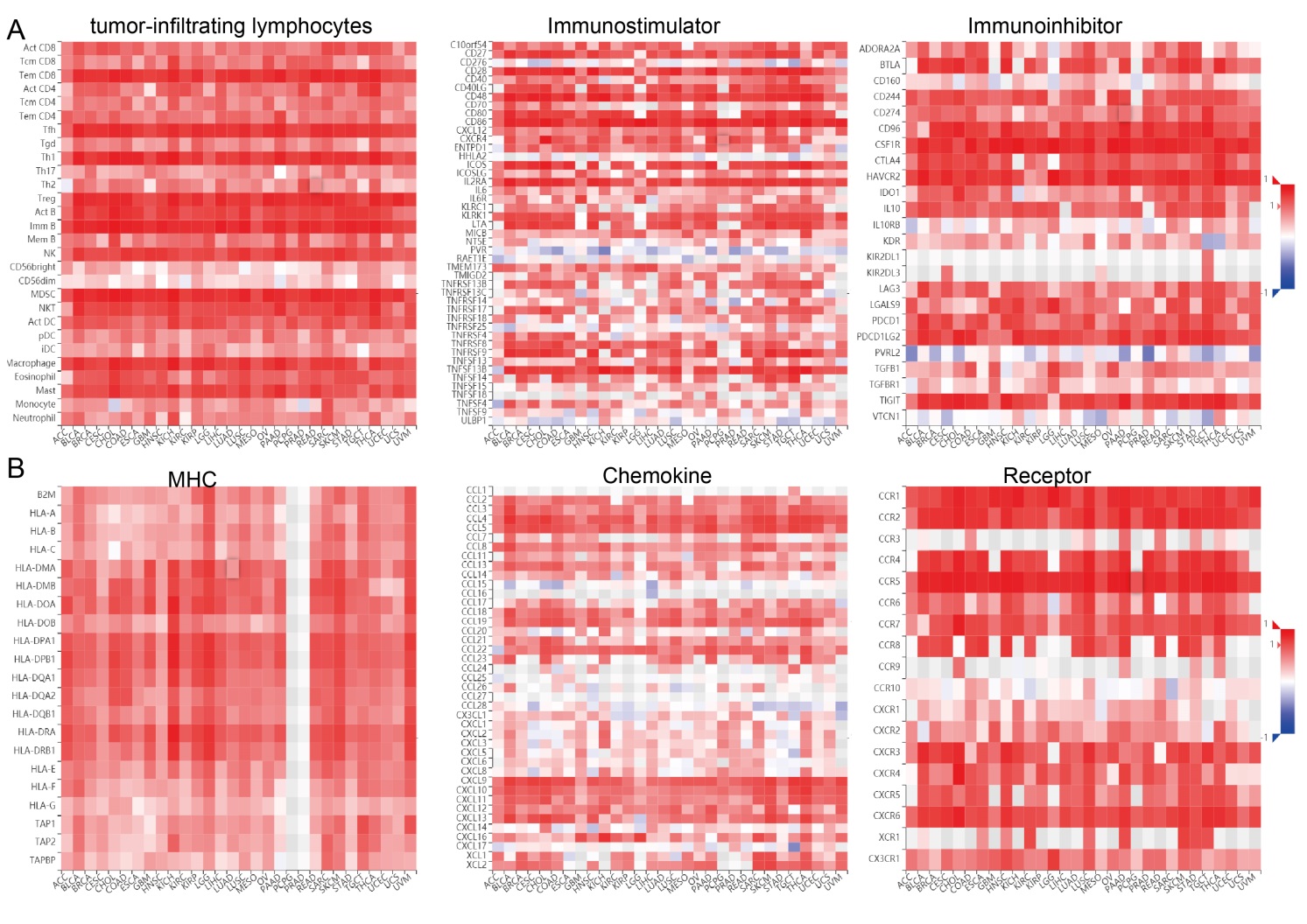


**Supplementary FIGURE 5 ⎜ Analysis the correlation between the PAFAH1B3 expression and diverse immune regulator.** (A)The correlation between the PAFAH1B3 expression and 28 tumor infiltrating lymphocytes analysis in pan-cancer by the TISIDB database. The correlation between the PAFAH1B3 expression and 45 immune-stimulator in pan-cancer analysis by the TISIDB database. The correlation between the PAFAH1B3 expression and 24 immune-inhibitor in pan-cancer analysis by the TISIDB database. (B) The correlation between the PAFAH1B3 expression and 41 chemokine in pan-cancer analysis by the TISIDB database. The correlation between the PAFAH1B3 expression and 18 receptor in pan-cancer analysis by the TISIDB database.The correlation between the PAFAH1B3 expression and 21 MHCs in pan-cancer analysis by the TISIDB database.


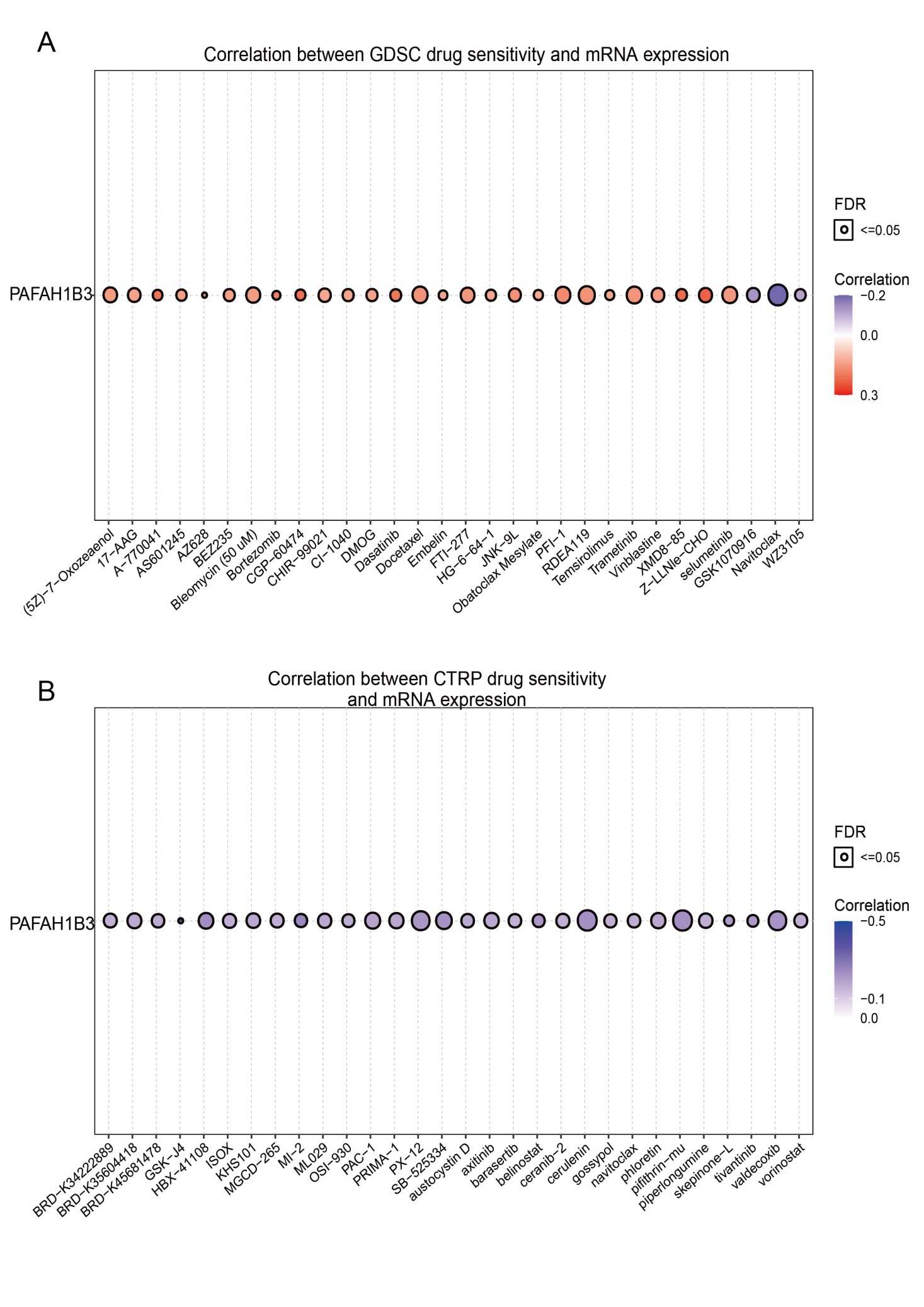


**Supplementary FIGURE 6 ⎜ Analysis the correlation between PAFAH1B3 expression and Drug sensitivity in diverse human cancer.** (A) The correlation between PAFAH1B3 expression and drug sensitivity in diverse human cancer analysis by GDSC database. (B) The correlation between PAFAH1B3 expression and drug sensitivity in diverse human cancer analysis by CTRP database. * P < 0.05, ** P < 0.01, *** P < 0.001.


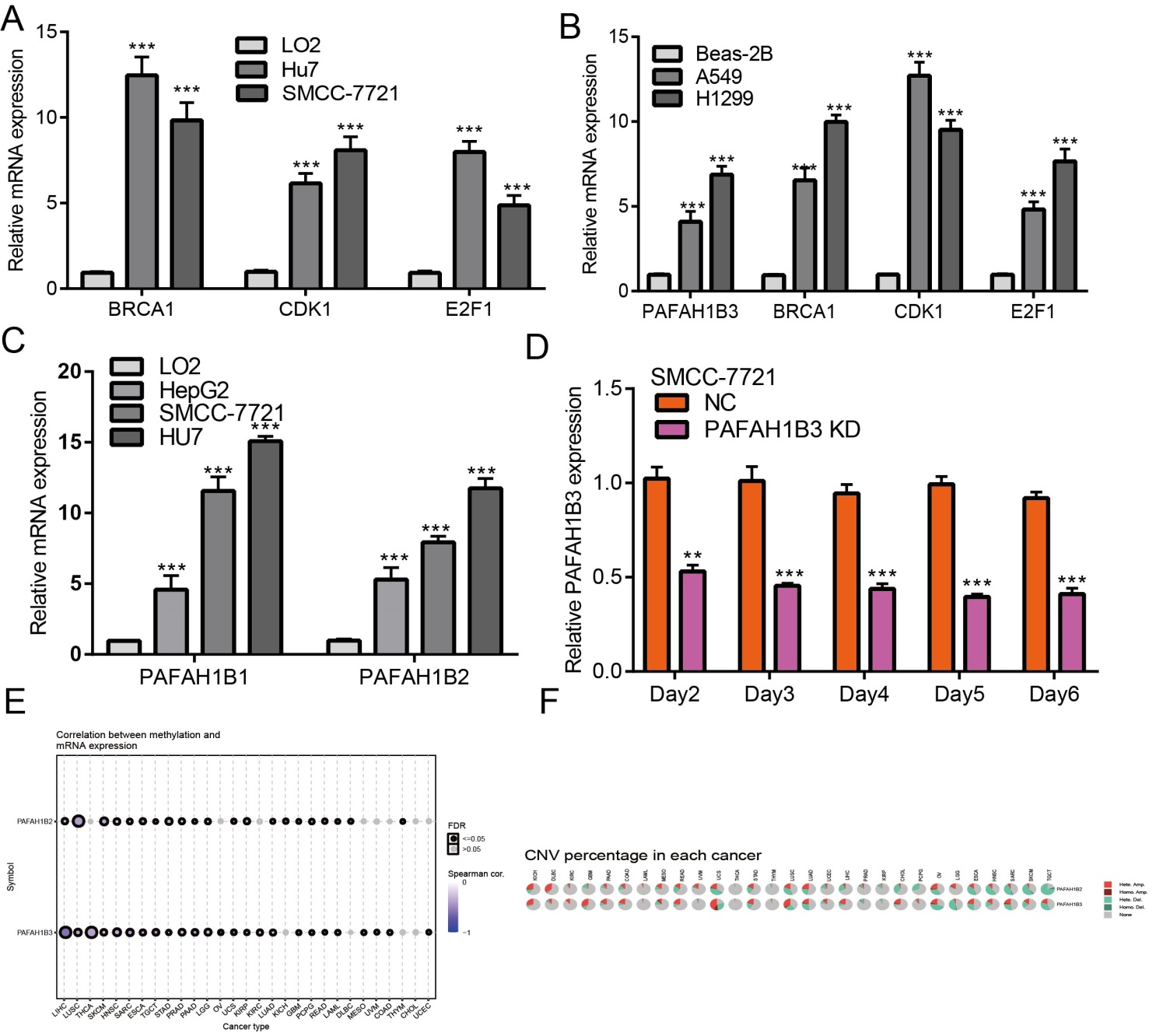


Supplementary FIGURE 7 (A-C) Analysis the expression of PAFAH1B1 and PAFAH1B2 and cancer hallmark genes in LIHC and NSCLC cells lines. (D) The PAFAH1B3 expression was gradually decreased in different time points examined by qRT-PCR assay. (E-F) analysis of DNA methylation and CNV of PAFAH1B2 and PAFAH1B3 in pan-cancers.


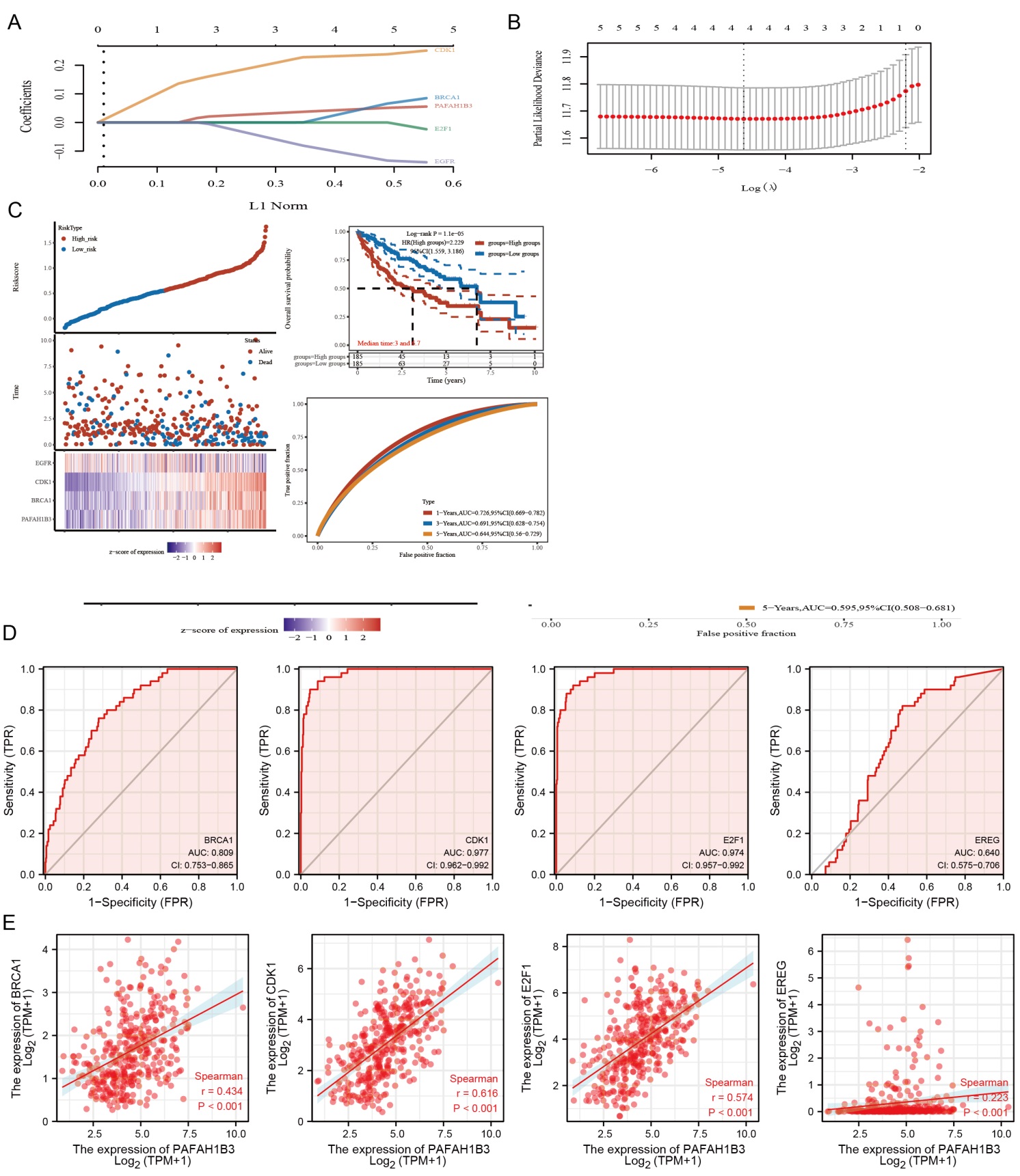


Supplementary FIGURE 8 (A-C) Using AFAH1B3, BRCA1, CDK1, E2F1 and EGFR to construct a prognostic gene model in LIHC. (D) ROC curve analysis of the AUC value of BRCA1, CDK1, E2F1 and EGFR in LIHC. (E)Correlation between AFAH1B3 expression and other cancer hallmark genes in LIHC.

**PAFAH1B2 promoter sequence**

Description: Homo sapiens platelet activating factor acetylhydrolase 1b catalytic subunit 2 (PAFAH1B2), transcript variant 1, mRNA. (from RefSeq NM_002572)

RefSeq Summary (NM_002572): Platelet-activating factor acetylhydrolase (PAFAH) inactivates platelet-activating factor (PAF) into acetate and LYSO-PAF. This gene encodes the beta subunit of PAFAH, the other subunits are alpha and gamma. Multiple alternatively spliced transcript variants have been described for this gene. [provided by RefSeq, Jan 2014].

GGGACTGCGGGGCTGGGGGAGGGATAGCATTAGGAGAAATACGGAATGTAAATGACGAGTTGATGGGTGCAGCAAACCAACATGGCACACGTATACCTATGTAACAAACCTGCACGTTGTGCACATGTACCCTAGAACTTGAATAAAAAAAAAAAAAAAAAAGAAAAATTACTTGACATATCTTGACATTTTAGGCTCCCAGGATTTTACAGTATTCTTAACAGGTTTTGTCAGCAAAATCAACCCCCACCCCCGCATCAGTTTAAGAGCCCTGGAAGGCTCTGCCTATTCACCAGCATAGAGCTGAGATCCTTAAAGGGCCAGTTCAGGGCACTGCTCCAGCAACCGTTGCGCTGCTATAAGCTTGAGCACATCTCTTGCCCCTCCTCTCTACTTTTATAGATCCTGCATTTATTTAGCATATAATCTTTTCAATATTGACTTTGTATATGTATGTCTTCCCCTAAACTGGATGTATCTTTTAAGGAAAAGCCTGGGGTTCGTCATCCCCCTATGTATCACCTTTACAGGCGGTCACCCAAAATCTCCACATTCATCATAGAAACAGAAATTCCACTAATAAATGTCAGGTACTGCCTCCTTAACCTTGGTCCGCTATTTCCCTCCCACTTTCAGCAGTCTGGTTTATTCCATGGGTGGAAGAGGAAGCCAAGTTGGCTGAGTGTGAAGGGAATGGCGTCAGTGTGACTTCACGCCACATCGTCCCGAATGCCAGCTCCACACTGCGGTGGCACGAACGCAGGATTGAGAGATGCAGCTGGAGTAAAGAGAGAAGCCCATGTCCCTCAGACGCCACACAAAATGGGGGTAACCGCGAGAACAAAGCAGGGTACCCAACGGGGTGTGGCGGGACACCTGGGCGCACGCCGCCAGCGCCCGGGAGAGCGAAGGCGGGGAGAGAGGAACCACGCTGAGGCGTGGCAGGATGGGGGCGGGACTTCCGGAAGCCATTTGGCTGAGTGGCAGGGGAACCGGAAGT

**PAFAH1B3 promoter sequence**

Description: Homo sapiens platelet activating factor acetylhydrolase 1b catalytic subunit 3 (PAFAH1B3), transcript variant 3, mRNA. (from RefSeq NM_001145940)RefSeq Summary (NM_001145939): This gene encodes an acetylhydrolase that catalyzes the removal of an acetyl group from the glycerol backbone of platelet-activating factor. The encoded enzyme is a subunit of the platelet-activating factor acetylhydrolase isoform 1B complex, which consists of the catalytic beta and gamma subunits and the regulatory alpha subunit. This complex functions in brain development. A translocation between this gene on chromosome 19 and the CDC-like kinase 2 gene on chromosome 1 has been observed, and was associated with cognitive disability, ataxia, and atrophy of the brain. Alternatively spliced transcript variants have been described. [provided by RefSeq, Mar 2009].

Gencode Transcript: ENST00000538771.5

Gencode Gene: ENSG00000079462.8

CTGTGTCTGGATCTGTCTCCCTAAACACATTGGGACAAGGTCAGGAGCTGACCCACTCATTTACTACTCATTCAGCAAGCACTTGTGGAATAACCACTGTGTGCCCGGCCCAGTTCCAGGAAAGTGGATTGAAAACATAAACACAATGAATAACTCATCTCTGTCTCAGTATCAATCCACCTCGAAGGAATGAATACATGGGAAGGTCCCACCTGGAGTGAGGCTACAAGAACATGACCAGGACGTTTAGCAGGGAGTGAGCCCCCCACCTGGTCCCAGAGAAGAGGGAGATCTGAGAAGACACTGAGGTCTGAGATTAATTTGGGGCAGCGTTGGGGGAGAGGCGCGACCCTAGGTGGCCTTGGCTATGGATGTAGGTTTCAGAGCTCAGACATACCCAGTTGGCCTGTGGGTTTAGAGAGAGTGTAGAGGCTGCAGATCTGCAAGATGATCTGCCGGAGGGACGCCAGACTGGACCTGAGCCCTGGAGGCGTCTCTAATTTCCTTTCTCTTTCTGCACCTTCTTCTACCCTCCAAGCAGGTCCTATGGTGTCTTGAATGCAAAGATATGTAGTTTATATGTTATGCCACGCGCGCGCACAACACACGGTGTTTTGAGTAGGGGTCCTGTGATGTCTTGAATGCAAAGATATGTACTTTAGATTTCAACACACACACACACACACACACACACACACACACACACACACCCACGGTGTTTTGAGTAGGGGAAGCCCACGAGTAGATAGGCTCACTAAAGCCCTGGCGCGGCCTTAGAGTGGCTCCTTAGAAGGGAGCCTCGAATTTCACATTTTGTCATCTTCCTTTTCTGTCCCCCACATCAGTAGAAAATAGGAGCGTCCCCCCCAGCCAGGTGCATTCGAGCCACCCCTTCAAGGGAATATAAGAATGGACTCGTCCACTCGCTCCCCTCCCCCCACCACGTGAATGGGTCACCCCCCCCCCCGCAACAGTAGGTGGTCCCTTCCGCACATTTGCTGTTTCCTGCTTTATGGTCGAACCTGTCTGGGTATCTGGGTACCACCATTAGTCCTTATCCTGATCTGTGACCCTGCAGTTCTGCAAGGTGTTTTTT
